# Emerging Functions of Regulatory T Cells in Tissue Homeostasis

**DOI:** 10.3389/fimmu.2018.00883

**Published:** 2018-04-25

**Authors:** Amit Sharma, Dipayan Rudra

**Affiliations:** ^1^Academy of Immunology and Microbiology, Institute for Basic Science (IBS), Pohang, South Korea; ^2^Division of Integrative Biosciences and Biotechnology, Pohang University of Science and Technology (POSTECH), Pohang, South Korea

**Keywords:** immune tolerance, autoimmunity, regulatory T cells, regulatory T-cells, Foxp3, tissue Treg, tumor Treg, regeneration

## Abstract

CD4^+^Foxp3^+^ regulatory T-cells (Tregs) are a unique subset of helper T-cells, which regulate immune response and establish peripheral tolerance. Tregs not only maintain the tone and tenor of an immune response by dominant tolerance but, in recent years, have also been identified as key players in resolving tissue inflammation and as mediators of tissue healing. Apart from being diverse in their origin (thymic and peripheral) and location (lymphoid and tissue resident), Tregs are also phenotypically heterogeneous as per the orientation of ongoing immune response. In this review, we discuss the recent advances in the field of Treg biology in general, and non-lymphoid and tissue-resident Tregs in particular. We elaborate upon well-known visceral adipose tissue, colon, skin, and tumor-infiltrating Tregs and newly identified tissue Treg populations as in lungs, skeletal muscle, placenta, and other tissues. Our attempt is to differentiate Tregs based on distinctive properties of their location, origin, ligand specificity, chemotaxis, and specific suppressive mechanisms. Despite ever expanding roles in maintaining systemic homeostasis, Tregs are employed by large varieties of tumors to dampen antitumor immunity. Thus, a comprehensive understanding of Treg biology in the context of inflammation can be instrumental in effectively managing tissue transplantation, autoimmunity, and antitumor immune responses.

## Introduction

Vertebrate immune and nervous system are two systems which are cognitive and under continuous interaction with the environment. This probably explains why both share common paradigms like recognition, learning or modulation, and memory. For long, immunology has been defined as a science of “self/non-self” discrimination (1). However, overtime, the immunological concept of “self” has evolved, where the very definition of an individual with defined anatomic borders, compatible balance between its parts, physiological autonomy, and ability to replicate as a unit is rapidly challenged by symbionts. Immune identity is now considered more fluid than restricted in strict borders (2). Hence, apart from generating a protective and offensive backdrop, the immune system must work to maintain an organismal identity by mediating dynamic exchange processes with the environment. This not only entails to generate robust defense against pathogens and toxins but also makes it rather paramount to suppress an overzealous immune response, curb autoimmune reactions, and maintain equilibrium toward commensals and food. Several mechanisms like clonal deletion, editing, anergy, ignorance, and immune deviation have evolved to safeguard against self-directed immunity (3). Specialized cells with immunosuppressive capabilities like tolerogenic dendritic cells (DCs) (4–6), regulatory B cells (7, 8), regulatory innate lymphoid cells (9), type 1 regulatory (Tr1) T-cells (10), and Foxp3^+^ regulatory T-cells (Tregs) (11–13) have also evolved.

Regulatory T-cells are arguably the most versatile immunosuppressive cells and work like immunological sentinels across various tissues. Both in mice and men, loss of these cells essentially results in breakdown of tolerance and multi-organ autoimmunity. Since their discovery, biology of Tregs has been a most dynamic field of immunological research and as a result, Tregs, which were once considered as a homogenous immunosuppressive population, have been found to be highly adaptable and diversified cell type. Their heterogeneity is now appreciated in the context of origin, localization, differentiation, and mechanisms of immunosuppression. In the first part of this review, we will briefly discuss the events which put Tregs to the center stage of immune research, following which we will attempt to elaborate on the various layers of Treg heterogeneity especially pertaining to non-lymphoid and tissue-resident Tregs.

## Concept of “Dominant” Tolerance and the Emergence of Regulatory T-Cell Research

T-cell tolerance is pivotal for regulating adaptive immune responses as T-cell help is essential for mounting an antibody response *via* B cells (14). T-cell tolerance for long, was studied in light of “recessive tolerance,” wherein T-cells with high affinity TCRs toward self-antigens are clonally deleted (15), or undergo “receptor editing” in thymus (16, 17). The runaway cells which escape these central processes encounter anergy or activation induced cell death in the periphery (15, 18). However, studies on tolerance ushered into an “active” or “dominant” era with the seminal discovery of suppressive CD4^+^ T-cells expressing high levels of high efficiency α-chain receptor of IL2 (CD25) (19).

### The Outset of Treg Research

Preliminary evidences of suppressive cells maintained in thymus started emerging when several investigators reported that neonatal thymectomy (3 day postnatal, 3dTx) could induce various autoimmune diseases in suitable mouse strains (20–25). Even more astonishing was the fact that similarly induced disease processes in rats could be reversed by reconstitution with normal lymphoid cells (26). Several groups tried to identify specific markers to distinguish suppressive cells from pathogenic T-cells in the thymus. It was reported that T-cells depleted of CD4^+^CD5^hi^ cells induced autoimmune phenotype akin to 3dTx in BALB/c and C3H mice (27). Two other groups demonstrated the capability of CD4^+^CD45RB^hi^ T-cells in inducing inflammatory bowel disease in BALB/c SCID mice (28, 29) and its resolution upon reconstitution with total T-cells. While these studies demonstrated that phenotypically distinct subsets of T-cells are capable of mounting discrete immune responses, specific identity of tolerance inducing counterparts remained elusive. Sakaguchi et al. in 1995 (19) discovered high surface expression of CD25 on about 8–10% of CD4^+^ T-cells, which were both CD5^hi^ and CD45RB^lo^ in concordance with previous studies. Asano et al. (30) demonstrated that CD4^+^CD25^+^ T-cells appear around day 3 postnatal and increase up to the adult levels by day 10. These authors were the first to propose the term “regulatory” for this subtype.

### Discovery of Foxp3

While subsequent studies involving numerous experimental models of autoimmunity established its functional existence (31), the usage of CD25 as a marker for Tregs remained controversial for a number of years due to its upregulation in all activated T-cells. Furthermore, it seemed possible that a subset of the activated T-cells, by virtue of marked upregulation of the IL2 receptor α on their surface, restrained immune response simply by competing for IL2.

A mouse line dubbed “scurfy,” with spontaneous autoimmunity (originally appeared as a spontaneous mutation at the Oak ridge national laboratory, USA under the Manhattan project), was immunologically characterized in 1991. Scurfy mice have an X-linked recessive mutation which leads to scaly skin, lymphoproliferation, hypergammaglobulinemia, lymphadenomegaly, anemia, runting, and early death (32). Thymectomy reduced the severity of the disease but did not totally ameliorate it. However, crossing the strain with *nu/nu* mice totally prevented the disease, suggesting thymic origin of disease causing cells. Several other studies revealed scurfy to be mainly a T-cell dependent disorder (33–35) much similar to Cytotoxic T-Lymphocyte Associated Protein 4 (CTLA4) (36) and Transforming growth factor β1 (TGFβ1) deficient animals (37). These similarities instigated investigations to identify the gene responsible for scurfy phenotype. In 2001, Brunkow et al. (38) identified 20 putative genes in a 500-kb region of X-chromosome by sequencing four overlapping bacterial artificial chromosomes. Out of these, one possessed an ORF highly homologous with DNA-binding domain of the forkhead/HNF3/winged helix family of proteins. This gene in scurfy mouse was found to harbor a 2-bp insertion mutation, resulting in a truncated gene product, deleting the C-terminal forkhead domain (38). Investigators designated this gene as *Foxp3*. Functional complementation experiments by mating scurfy carrier females with *Foxp3* transgenic lines resulted in complete rescue of the scurfy phenotype, corroborating *Foxp3* mutation as the cause (38).

At around same time, mutations in *FOXP3* gene and its 3′ untranslated region were confirmed in human patients of IPEX syndrome (39, 40). IPEX syndrome is immunodysregulation polyendocrinopathy enteropathy X-linked, originally described in 1982 by Powell et al. (41). The striking similarity in autoimmune phenotype of IPEX patients, scurfy and 3dTx mice led several groups to examine the function of *Foxp3* in Tregs. Subsequently, in 2003, three studies reported that indeed *Foxp3* is uniquely expressed by CD4^+^CD8^−^CD25^+^ thymocytes and CD4^+^CD25^+^ peripheral regulatory T-cells (42–44) in mice. Retroviral transduction of *Foxp3* induced CD25 expression in CD4^+^CD25^−^ T-cells which were functionally suppressive and expressed Treg associated molecules CTLA4 and GITR. Deletion of *Foxp3* in mice resulted in lymphoproliferative disorder identical to scurfy mice (43, 44). Mixed bone marrow chimera experiments demonstrated that indeed only *Foxp3*-sufficient bone marrows were capable of generating CD4^+^CD25^+^ Tregs (44). Conclusive evidence for Foxp3 as lineage specific marker for mice Tregs came from Foxp3^eGFP^ reporter mice (45) in which GFP expression was found only in TCRβ^+^ T-cells among all hematopoietic cellular compartments. Conditional deletion of *Foxp3* in CD4^+^ T-cells led to a lymphoproliferative disorder mirroring scurfy phenotype. These series of experiments established Foxp3 as the molecular identity responsible for implementing Treg transcriptional signature. Further investigations revealed that the *Foxp3* gene itself is regulated by three conserved non-coding sequences (CNS) 1–3. Detailed epigenetic analyses have identified CNS1 as the TGFβ responsive element which is required for peripheral generation of Tregs, CNS2 is involved with heritable maintenance of Foxp3 expression while CNS3 acts as a pioneering element for thymic induction of *Foxp3* (46). Proteomic analyses demonstrated Foxp3 to be interacting with more than 350 proteins in multiprotein complexes, many of which are transcription-related factors (47). A detailed review on regulation of *Foxp3* and Foxp3 mediated regulation of the Treg transcriptome can be found in Lu et al. (12).

## Developmental and Phenotypic Diversity in Tregs

Neonatal thymectomy experiments in mice confirmed beyond doubt that early development of Tregs happens in thymus. A detailed discussion on thymic development of Tregs can be found in Ref. (13, 48, 49). Briefly, cell surface markers indicative of strength of TCR interaction (CD25, CD5, etc.) suggested the involvement of TCR signaling. TCR repertoires of Tregs have limited overlap with that of non-Tregs and are largely self-reactive (50). Nur77-GFP reporter mice which express GFP under *Nur77* gene locus, an early gene expressed upon TCR stimulation have higher GFP expression in thymic Tregs (tTregs) (51). With regard to cytokines, it was reported earlier that mice lacking either IL2 or CD25 (45) are able to generate Tregs, albeit at a reduced level. However, if common γ chain is deleted, Tregs are not formed (45). This suggests cooperation among γ chain cytokines in *Foxp3* expression and maintenance. Thus, the current model of Treg generation in thymus gravitates toward an instructive one wherein TCR signaling substantially above the strength required for positive selection and relatively near the strength that induces negative selection initiates specification toward pre-Treg state. In the second step, cytokines induce *Foxp3* expression. More recently, Satb1, a genome organizer and transcription factor was shown to at least partially mediate the genomic arrangement of super-enhancers responsible for Treg development (52). The Treg specific super-enhancer patterns were found “poised” for activation even in conventional peripheral T-cells.

Though thymic regulatory T-cells are adept at suppressing autoimmune responses against self-antigens, a reasonably tolerant immune environment cannot be developed if repeated immune responses are mounted against beneficial and innocuous microbes as well as food antigens. In part, this is achieved by generation of Tregs in the periphery. Indeed, initial evidences suggested that Tregs can be generated by oral antigen feeding (53, 54) as well as by antigen-specific APCs (55) in the absence of functioning thymus. Also, conventional CD4^+^CD25^−^ naïve T-cells could be converted to CD4^+^CD25^+^CD45RB^−/low^ suppressor cells by costimulation with TCR and TGFβ. TGFβ activates Smad2 and 3 transcription factors (56) which redundantly, help in peripheral Treg generation by initiating a cascade of interactions with specific enhancer regions within the *Foxp3* locus (56–58).

Contrary to initial interpretations of Tregs being a universal immunosuppressive population, diligent interrogations led to identification of a rather diverse and distinct pool of heterogenous subsets. Other than the site of induction, Tregs were classified into two separate populations: central Tregs (cTregs) and effector Tregs (eTregs) (59, 60). cTregs are comparatively quiescent Tregs in the lymphoid tissues. They express the lymphoid homing molecules CD62L and CCR7 and are dependent on IL2 secreted by Tconv in T-cell zones of lymphoid tissues (60). On the other hand, eTregs are primarily non-lymphoid Tregs which downregulate lymphoid homing molecules and upregulate CD44, ICOS, GITR, and other activation-induced markers. For maintenance, they are dependent on sustained ICOS signaling (60). Most of these cells express transcription factor BLIMP1 and produce high IL10, akin to a population of ICOS^+^IL10^+^ Tregs in humans (61).

Investigations into Treg mediated suppression of distinct helper T-cell immune responses imply a contextual T helper–Treg coupled viewpoint of Treg heterogeneity. It was reported that Tregs express high amount of the interferon (IFN) regulatory factor IRF4, Treg specific ablation of which resulted in selective Th2 related pathologies (62). These findings initiated similar investigations in other helper T-cell contexts and indeed, now a well-established paradigm exists wherein, in a Th1 inflammatory context Tregs express transcription factor Tbet, responsible for Th1 speciation, and express CXCR3 to accumulate at such sites (63). Transcription factor STAT3 is expressed in Tregs in a Th17 context (64) which helps in upregulation of CCR6 to migrate to intestine and production of IL10 (65). Similarly, Bcl6 expression in Tregs was shown to be important for regulating Tfh cells and expression of CXCR5 (66, 67).

Moving a step further and increasing complexity and diversity among Tregs, several Treg subpopulations are discovered in non-lymphoid tissues. These Tregs are not only found to be instrumental in suppression of inflammatory responses but also integrate into a larger biological paradigm for the benefit of organ and organismal homeostasis. In the following sections, we will review the characteristics of non-lymphoid Tregs, how they originate and function in maintaining homeostasis of tissues. We will attempt to elucidate if, apart from location, these cell types can be further identified by virtue of tissue-specific phenotypic and functional characteristics. We will elaborately discuss the well-characterized Treg populations in adipose tissues (AT), intestines, and skin, as well as attempt to highlight upon some of the recently identified ones in other tissues like muscles, lungs, and placenta. While overzealous Tregs in malignancies not necessarily can be clubbed with normal tissue Tregs, but at certain level, as Tregs perceive the context of the environment they reside in and are hijacked by tumors for their benefit, we will also discuss tumor infiltrated Tregs in order to elucidate on the general principles that might integrate such heterogeneity.

## Fat Tregs: Immunosuppression for Metabolic Homeostasis

Adipose tissue is natural calorie reservoir of the body. Overall, the tissue is classified as two somewhat functionally antagonistic tissue types: white adipose tissue (WAT) and brown adipose tissue (BAT). WAT stores the excessive nutrients in the form of fat droplets during over-nutrition and releases it under energy deficit conditions. BAT, by virtue of higher expression of the uncoupling protein 1 (UCP1) protein, enhances energy utilization by non-shivering thermogenesis (68). WAT is abundantly available and found mainly in subcutaneous tissue, omenta, mesenteries, perirenal tissues, and bone marrow, while BAT has a restricted distribution occurring mainly in interscapular and inguinal regions.

### AT Architecture

Structurally, AT is a loose connective tissue comprising mainly of fat cells (adipocytes), each surrounded by its own basal lamina and separated by a thin layer of extracellular matrix composed of reticular and collagen fibers and supplied with numerous capillaries. Several other resident as well as transient cells are strewn around in AT namely fibroblasts, myofibroblasts, and immune cells like macrophages, mast cells, eosinophils, neutrophils, and T-cells. WAT are the professional fat depots (69) which store excess energy as triglycerides without the common lipotoxicity experienced by other cell types (70). WAT adipocytes are usually spherical with a single fat droplet occupying 90% cell volume and a thin elongated mitochondrion on one side. On the other hand, BAT integrates environmental conditions *via* brain adrenergic responses toward cold temperatures. Adipocytes in BAT are typically polygonal, containing triglycerides in multiple small vacuoles, and are characterized by numerous large, spherical mitochondria.

### Fat Treg Origin and Accumulating Factors

**Figure 1:**
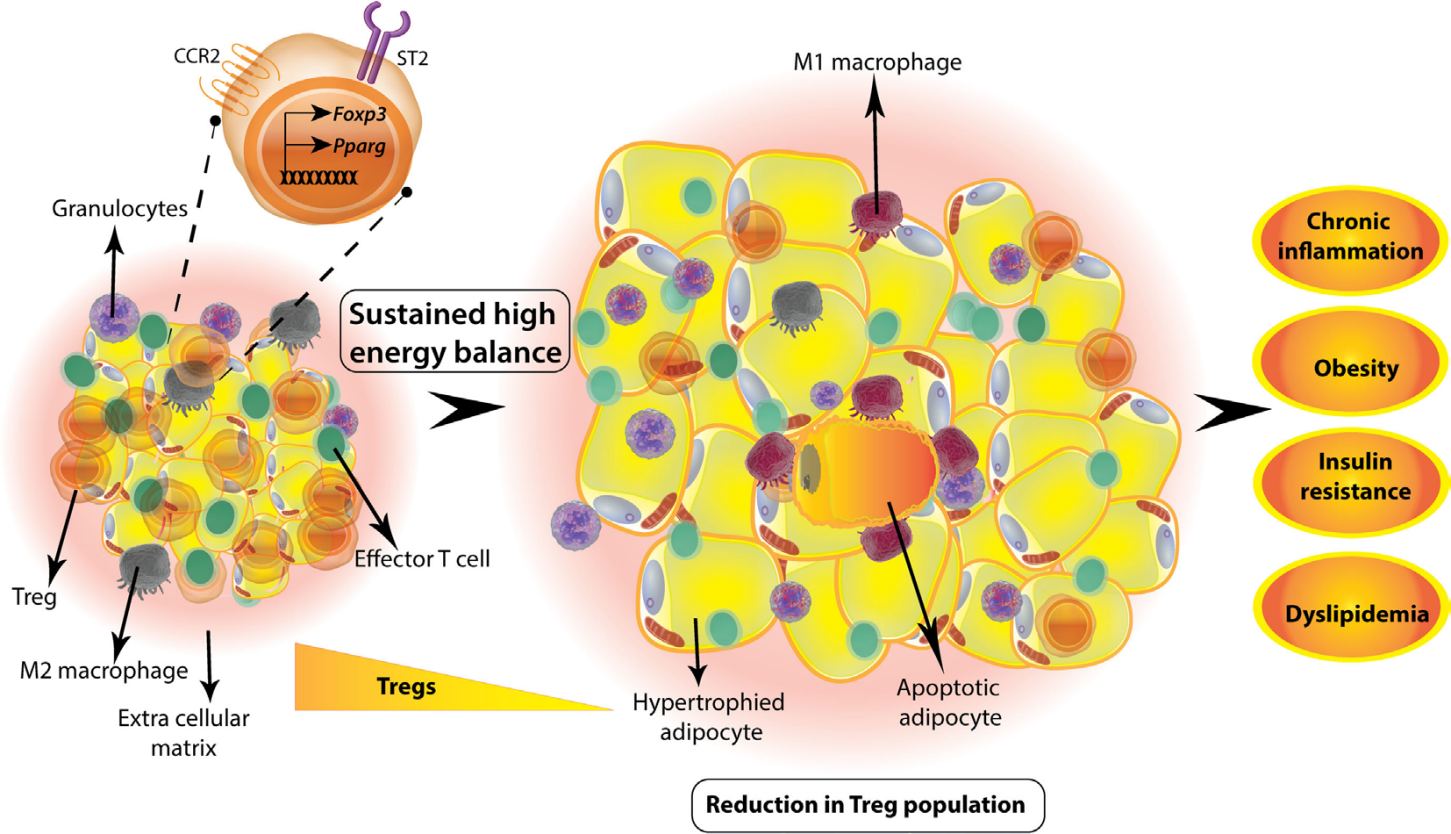
Visceral white adipose tissue (WAT) regulatory T-cells (Tregs) are involved in metabolic homeostasis. WAT Tregs adapt to adipose environment by expression of PPARγ that regulates genes involved in lipid metabolism. These Tregs also express alarmin IL33 receptor ST2 and adipose chemokine receptor CCR2. Tregs are abundant in lean adipose tissue of adult mouse; however, if there is a sustained positive energy balance as in high-fat diet-induced obesity animal model, then the Treg numbers decrease drastically. This is concomitant to a change in macrophage phenotype from anti-inflammatory M2 to inflammatory M1 macrophages and unhealthy hypertrophy of adipocytes. Sustained hypertrophy leads to adipocyte apoptosis and exacerbated inflammation. Overall, decrease in adipose Tregs is accompanied by obesity, insulin resistance, dyslipidemia, and chronic low-grade inflammation.

The plethora of adipokines and cross-talk with nervous as well as immune system has underscored the importance of AT as an endocrine organ with profound effects on body’s metabolic homeostasis. However, as adipocyte increase in size due to excess calories, hypoxia sets in leading to accumulation of inflammatory macrophages (Figure 1) in obese adipose (71). Subsequent upregulation of several inflammatory adipokines (72) can activate CD4^+^ T-cells independent of macrophages. Any immune response shall be regulated so that it does not outlive its utility; a task largely achieved by several anti-inflammatory immune cell types already resident in the adipose (73–77).

**Figure 2:**
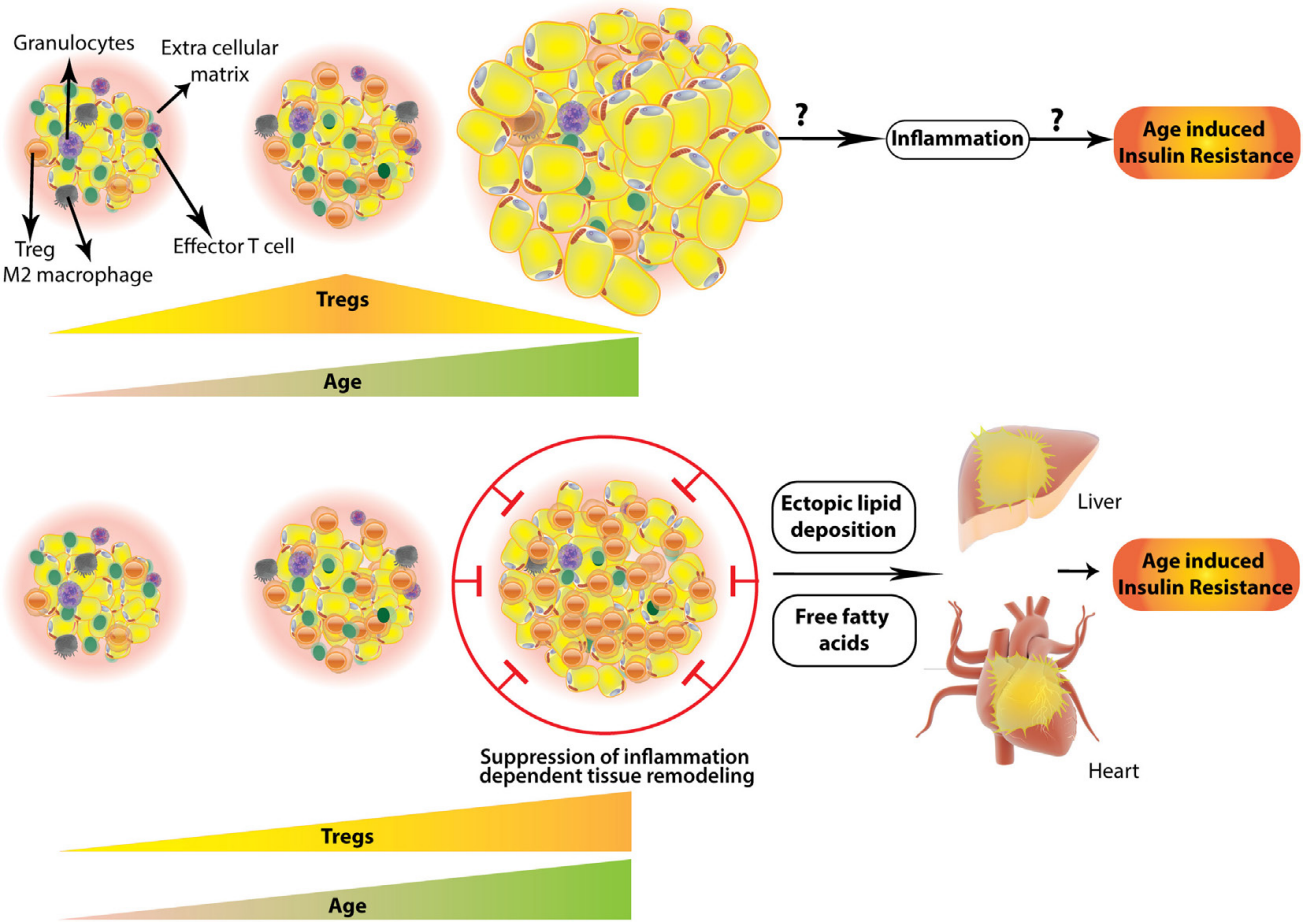
Two contrasting scenarios of regulatory T-cells (Treg) numbers and their outcome in aged white adipose tissue (WAT). WAT Tregs increase with age reaching a plateau and then decrease abruptly in aged (~45 weeks) mouse (top). Whether this results in inflammation leading to age-associated insulin resistance is not explored; however, (bottom part) contrasting evidence suggest that white adipose Tregs keep on increasing even in the aged adipose tissue, which in concordance with “adipose tissue expandability hypothesis” (see text) results in suppression of healthy inflammation required for remodeling of adipose. This results in a storage space problem leading to ectopic deposition of fat in visceral organs, like liver and pericardium. This is accompanied by free fatty acid induced lipotoxicity and age-associated insulin resistance.

In a seminal study, Feuerer et al. (76) identified unique fat-residing Tregs in mouse and human ATs. Surprisingly, unlike peripheral lymphoid compartment where normally 10–15% of CD4^+^ T-cells are Foxp3^+^ Tregs, almost half of the fat CD4^+^ T-cells are Foxp3^+^ Tregs, which were found to accumulate primarily in visceral fat over a period of time since birth, peaking at around 25 weeks of age. In one study, this characteristic accumulation of Tregs was found to be accompanied by a sudden drop to fifth week levels (78) in mice aged further, suggesting a negative correlation between the frequency of visceral WAT resident Tregs and age-related metabolic inflammation (Figure 2). In contrast to this finding, a recent study, reported even greater accumulation of Tregs in older mice, implicating an alternative viewpoint instead (79) (Figure 2). The cause of this discrepancy might be different colonies, husbandry practices, as well as microbial and dietary composition. Nonetheless, the aged visceral WAT Tregs were found to be functional (79).

As far as origin of AT Tregs are concerned, visceral WAT Tregs appear to be largely of thymic origin. A thymectomy beyond 3 weeks of life does not affect visceral WAT Tregs population in mice (80). TCRα sequencing experiments from “Limited” mice [mice with limited focused diversity restricted to CDR3α (81)] showed that TCR repertoire of fat Tregs are different from conventional fat T-cells. Furthermore, visceral fat Treg repertoire was only a restricted subset of lymph node (LN) Tregs TCR repertoire (76), suggestive of an abdominal WAT specific distinct TCR repertoire of Tregs. This indicates either a continuous supply of Tregs from peripheral LNs or an initial seeding of tTregs followed by selected clonal expansion in AT. Adoptive transfer of congenically marked Tregs confirmed that visceral WAT Tregs are not significantly derived from circulating Tregs (80). Also, very high expression of both Helios and Neuropilin1 (Nrp1) as well as transcriptomic analysis of visceral WAT Tregs suggest their thymic origin and little to no conversion of naïve T-cells into visceral WAT Tregs (80).

Origin of subcutaneous WAT Tregs and BAT Tregs have not been studied in much detail. Naïve T-cells isolated from both these tissues, however, produce significantly high number of induced Tregs than visceral WAT in *in vitro* conversion assays (82).

### Tissue Adaptation and Phenotype

That extralymphoid Tregs adapt to the context and microenvironment is best explained by higher expression of transcription factor PPARγ in fat Tregs (83, 84). Co-immunoprecipitation studies confirmed PPARγ interaction with Foxp3, suggesting a visceral WAT-specific Foxp3-PPARγ-mediated gene expression axis (83). Using a Blimp1-GFP reporter mouse, Vasanthkumar et al. (84) showed that most of the visceral WAT Tregs fall in the category of eTregs. For identifying the survival factors for eTregs, they reported that visceral WAT Tregs specifically express *Il1rl1*, which encodes alarmin IL33 receptor ST2. Both IL33 deficiency in general, as well as T-cell intrinsic ST2 deficiency resulted in specific reduction in number and percentage of visceral WAT Treg compartment (84). In *in vitro* settings, both IL2 and IL33 were able to induce proliferation and ST2 upregulation in a fraction of T-cells upon TCR stimulation which was dependent on MyD88 (84). Development and proliferation of visceral WAT Tregs appears to be dependent on two signals: (1) TCR crosslinking which induces PPARγ and ST2 expression *via* BATF and IRF4, both of which bind to the intronic regions of *Pparg* and *Il1rl1* genes (84) and (2) IL33 which *via* MyD88 feeds forward the expression of ST2 (84). In accordance to the concept of local adaptation, PPARγ in visceral WAT Tregs upregulates the expression of lipid metabolism genes like *Dgat1* (diacylglycerol *O*-acyltransferase 1), coding for an enzyme which catalyzes the terminal step in triacylglycerol synthesis by using diacylglycerol and fatty acyl CoA as substrates; and *Pcyt1a* (choline-phosphate cytidylyl transferase A), an enzyme involved in regulation of phosphatidylcholine biosynthesis (83). It is possible that these gene products enable visceral WAT Tregs to survive in a lipotoxic environment and/or enable the utilization of fatty acids as metabolic fuel. Other than these essential factors, visceral WAT Tregs also express high levels of GATA3, Klrg1, early activation marker CD69, and adipose signature chemokine receptor CCR2. BAT Tregs are found to be very similar to WAT Tregs at transcriptional level with higher expressions of PPARγ, IL10 and chemokine X ligands 1 and 2 (85). The under-expressed transcripts were those encoding for TCR signaling specific T Cell Factor 7 and cytokine IFNγ (85).

### WAT Tregs Are Important Players in Metabolic Syndrome

Adipose originated pro-inflammatory cytokines, like TNFα, IL6, and type1 IFNs, have been suggested as causative of insulin resistance and metabolic syndrome (86–88). Also, human obese subjects were found to be deficient in circulating Tregs, whose levels were inversely correlated with body weight and BMI (89). Considering the primarily immunosuppressive phenotype of Tregs, it is expected that fat Tregs play a major role in controlling adipose inflammation, and in turn, affect the overall metabolic homeostasis of the body. Indeed, in Foxp3^DTR^ mice, in which the gene encoding diphtheria toxin receptor (DTR) is knocked into the *Foxp3* locus (90), Treg depletion upon diphtheria toxin (DT) administration, leads to visceral WAT tissue inflammation (76). However, total Treg deletion also initiates a strong systemic inflammatory response (90). Hence, a visceral WAT specific model for Treg deletion was required. This was achieved by Treg specific deletion of PPARγ, which resulted in more than 80% reduction in visceral WAT Tregs, without any effect on splenic Treg population (83). This decrease in Treg population was accompanied with marked inflammatory cell infiltration in visceral WAT (83). In a high-fat diet-induced obesity model, WAT Treg numbers are reduced drastically (Figure 1), which can be rescued by treatment with a synthetic PPAR ligand pioglitazone, which improves insulin sensitivity by working on PPARγ1 and 2 and affecting lipid metabolism (91). Its administration was able to improve the metabolic parameters in wild-type mice but not in mice harboring PPARγ-deficient Tregs, suggesting a direct role of PPARγ expression in visceral WAT Tregs.

Interestingly, the role of visceral WAT Tregs in aging animals has been reported to be opposite to what was observed in comparatively young animals. Contrary to obese animals, depletion of visceral WAT Tregs in aged animals improved the metabolic parameters and rescued aging induced insulin resistance (79) (Figure 2). Treg specific deletion of PPARγ led to less increase in fat and more in lean weight with age (more than 45 weeks). The adipocyte size was less and hepatic triglyceridosis was decreased. An increase in total Tregs by IL2–IL2 antibody immune complex resulted in reduced glucose uptake by aged adipocytes, suggesting compromised storage function (79). Similar results were obtained upon external IL33 administration. Overall this study reveals an unexpected cooperative role of visceral WAT Tregs in age-associated insulin resistance and metabolic inflammation. One explanation of this seemingly counter-intuitive finding might be extended by the so called “adipose tissue expandability” hypothesis. This hypothesis posits that in a state of “positive energy balance” metabolic complications arise because WAT is not able to expand further and accommodate excess calories, essentially propounding that metabolic syndrome coming out of excessive nutrition and obesity is actually a storage space problem (92). Indeed, animal models where AT inflammation is controlled result in decreased AT hyperplasia in a high-fat diet-induced mouse model of obesity (93), which causes ectopic lipid depositions (hepatic steatosis, dyslipidemia, etc.) and worsened metabolic parameters (93). Also, obese ob/ob mice which were made transgenic for full-length adiponectin and thus had adiponectin levels equivalent to treatment with a PPARγ agonist, showed uninhibited WAT expansion leading to morbid obesity but improved insulin sensitivity and other metabolic parameters (94) owing to reduced ectopic lipid deposition in liver and muscles (94). Given these observations, whether and how age-related accumulation of the visceral WAT Tregs results in compromised AT hyperplasia, remains to be seen.

### BAT Tregs Help in Thermogenesis

A generalized Treg ablation alters the metabolic profile of mice with regard to BAT as well. DT mediated deletion of Tregs in Foxp3^DTR^ mice resulted in reduced whole-body oxygen consumption in a short-term cold temperature exposure model (85). That the Tregs are at forefront of metabolic homeostasis and their interventions are highly context dependent is further strengthened by a recent study analyzing BAT Tregs in detail. While a short-term (2 weeks) high-fat high-sugar (HFHS) diet, promoting thermogenesis (95), increased BAT Tregs, it actually decreased visceral WAT Tregs in young adult mice (82). On the contrary, a long-term HFHS diet (16 weeks) significantly reduced visceral WAT Tregs but made no impact on percent of BAT Tregs. This suggests that the tenor of caloric intake can have specific effect on tissue Tregs. In accordance to the role of BAT Tregs in non-shivering thermogenesis, treatment with ADRB3 (β-3 adrenergic receptor) agonist increased Tregs in BAT. However, “betaless” mice which are deficient in all three (β-1, 2, and 3 adrenergic receptors) did not have a reduced percentage of BAT Tregs, suggesting a redundant role of adrenergic signaling in BAT Treg accumulation *per se*. Treg depletion, followed by β-3 stimulation on the other hand did result in reduced levels of BAT thermogenic and lipolytic genes (*Ucp1, Ppargc1a, Pparg, Prdm16, Lpl*, etc.) confirming Treg functionality in BAT thermogenesis (82). It will be interesting to know if this results in reduced metabolic adaptation in cold exposure. Also, whether these effects are intrinsic to Tregs can only be ascertained with Treg specific *Adrb3* deletion. Thus, these studies confirm a role of Tregs in BAT which goes beyond immunosuppression and actively associates Tregs with cold adaptations of the body by regulating lipolysis and thermogenesis.

## Intestinal Tregs: Preserving the Holobiont

The mammalian digestive system performs two very vital functions—digestion and absorption of food and shaping a gut microbial ecosystem. According to recent estimates, human colon harbors about 4 × 10^13^ bacteria (96, 97), a density (10^11^/mL) highest among any microbial habitat (98). It has a perplexing task to efficiently implement a “goldilocks” balance between two seemingly opposite events; permit absorption of nutrients but check exposure to harmful substances, guard against invasive pathogens but facilitate colonization of commensals and help them thrive.

### Architecture

Incessant provision of food and microbial antigens has resulted in typical structural adaptations in gut (99, 100) and evolution of specialized gut immune cells for immunosurveillance and maintenance of tolerance (101). The small intestine (duodenum, jejunum, and ileum) with maximum absorptive surface created by large circular folds (plicae) and finger like projections (villi and microvilli), has evolved as the primary organ for food absorption. The folding results in formation of deep invaginations, crypts of Lieberkühn, which house Paneth cells that secrete antimicrobial molecules upon exposure to bacterial antigens. Several enteroendocrine cells and mucus producing Goblet cells are also interspersed among small intestinal epithelial cells. The large intestine (cecum, colon, and rectum) harbor majority of commensals and perform the vital functions of absorbing water and vitamins while converting the undigested food into feces. The large intestinal walls are protected from luminal contents by two layers of mucus, the outer (luminal) thin mucus layer which hosts most of the bacteria, and the inner thick mucus layer which is largely sterile (102, 103).

Histologically, intestinal tract contains four layers—mucosa, submucosa, muscularis propria, and adventitia or serosa. The mucosa is the layer where most of the immune processes take place. It consists of an epithelial layer, which has scattered intraepithelial lymphocytes (IELs), underlying lamina propria (LP) and a thin muscle layer muscularis mucosa. The LP consist of non-cellular connective tissue, like collagen and elastin, blood and lymphatics, myofibroblasts, and nerve endings, and is densely packed with immune cells including mononuclear cells, plasma cells, B and T lymphocytes including Tregs, eosinophils, macrophages, and mast cells (104).

Different parts of intestine drain into separate LNs, like duodenum drains into a LN embedded in pancreatic tissue; mesenteric LNs drain jejunum, ileum, cecum, and ascending colon; two small LNs in pancreatic tissue drain transverse colon and descending colon and rectum primarily drains to caudal LNs (101). Anatomic variations largely define the antigenic variations of intestine as well. While small intestine grapples for equilibrium against food antigens, large intestines have an overwhelming load of microbial antigens. As much as these are foreign to the body, they are equally essential. Thus, for the economy of immune response it is highly desirable to assimilate those in the immunological self. The intestinal population of Tregs is arguably the most important cell type to contain the immune response against both food and innocuous microbial antigens and maintenance of intestinal homeostasis (105, 106).

### Origin of Intestinal Tregs

Like other non-lymphoid tissues, Tregs are also enriched in intestinal LP with colon harboring 25–35% (107–109) and small intestine harboring 10–15% (109, 110) Tregs among total CD4^+^ T-cells. Apropos to the prevalent inflammatory milieu and antigenic environment, the colonic Tregs are largely developed against microbial antigens. GF mice, devoid of any microbiota, have several folds less number of colonic Tregs compared to specific pathogen-free (SPF) mice (111, 112). A long-term broad-spectrum antibiotic treatment also reduces colonic Tregs in SPF mice (111). As small intestine is seat for nutrient absorption, most of the small intestine LP (siLP) Tregs do not develop against bacterial antigens as evident by comparable number of Tregs in SPF and GF mice (109). However, once the GF mice are brought up as antigen-free (AF) mice by provision of only elemental diet post-weaning, there is a marked decrease in siLP Tregs (109). A subset of Tregs, however, remains in colon as well as small intestine of GF and AF mice, presumably specific to gastrointestinal self-antigens.

**Figure 3:**
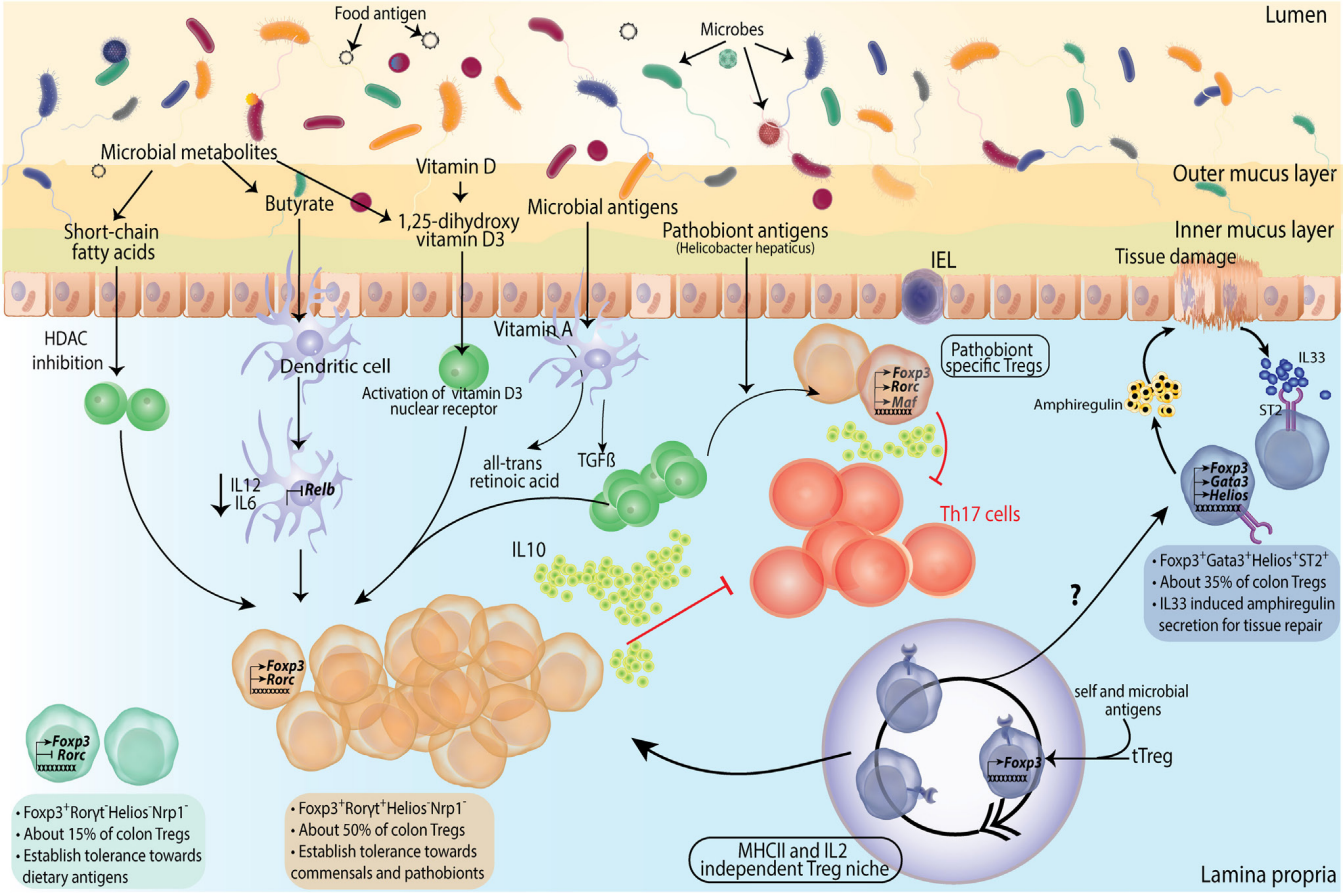
Subtype differentiation of regulatory T-cells (Tregs) in large intestine. Colonic Tregs differentiate into three different subtypes based on RORγt (Rorc) and GATA3 expression along with Foxp3. Foxp3^+^RORγt^−^ cells comprise ~ 15% of colonic Tregs, their function in colon is not much elucidated but presumably these are responsible for establishing tolerance to dietary antigens. Second and major subset of colonic Tregs is Foxp3^+^RORγt^+^ Tregs, constituting about 50% of total colonic Tregs. These cells are primarily involved in microbial tolerance and several mechanisms contributing to their generation has been described. From left, short chain fatty acids generated by microbial fermentation can differentiate naive T cells into Foxp3^+^RORγt^+^ Tregs in lamina propria (LP). Butyrate, specifically, can contribute to Foxp3 expression and function by virtue of its histone deacetylase (HDAC) inhibitory function, as well as can turn dendritic cells (DCs) into Treg generating tolerogenic cells by suppressing their IL12 and IL6 levels and expression of NFκB subunit Relb. Vitamin D metabolite 1,25-dihydroxy vitamin D3 differentiates naive T cells into Tregs by activating its nuclear receptor. TGFβ along with DC generated vitamin A metabolite all-trans retinoic acid differentiates naive T cells into Tregs in presence of microbial antigens. Foxp3^+^RORγt^+^ Tregs deploy various mechanisms toward establishing dominant tolerance with IL10 production and direct suppression of Th17 cells as prominent ones. A special subset of Foxp3^+^RORγt^+^ Tregs express cMAF and is instrumental in establishing tolerance to pathobionts in homeostasis. Foxp3^+^RORγt^+^cMaf^+^ Tregs are identified against specific epitopes of *Helicobacter hepaticus* and suppress epitope specific Th17 cells. It has been shown that in absence of pTregs, early seeding of thymic Tregs (tTregs) happens in colonic LP. These tTregs expand into a niche which is independent of MHCII and IL2 signaling but depends on microbial antigens. These Tregs subsequently fill up the LP Treg compartment. However, whether these can differentiate into all Treg subtypes is not known. A third subtype of Tregs express GATA3 (not RORγt) and constitutes about a third of total Tregs. These Foxp3^+^GATA3^+^ Tregs express ST2 receptor which bind to tissue damage-induced alarmin IL33. Foxp3^+^GATA3^+^ Tregs are instrumental in suppressing inflammation and facilitating tissue repair by secretion of amphiregulin upon tissue damage.

Sequencing of colonic Treg TCRs of genetically engineered mice with limited polyclonal repertoires found that TCR usage of colonic Tregs was different from that of other tissues (113). Also, there was very little similarity of TCR usage between Tregs and naïve or effector Foxp3^−^ T-cells (113). Stable chimeras made from retroviral transduction of colonic Treg TCRs to bone marrow progenitors resulted in induction of respective TCR expressing Tregs preferentially in colon while no specific TCR bearing cells were found in thymus (113). Adoptive transfer of naïve T-cells from the transgenic lines made from colonic TCRs resulted in very efficient conversion to peripherally induced Tregs (pTregs) in mesenteric LNs and colon (114). Other than the specific TCR repertoire, high surface expression of Helios, an Ikaros family transcription factor (115), and Nrp1, a membrane-bound co-receptor for vascular endothelial growth factor and semaphorin (116, 117), has been found to be associated with tTregs. Although the precise origin of Tregs based on these markers is debatable since their expression can be upregulated in inflammatory settings (118, 119), nevertheless, most of the studies have reported enrichment of Helios^−^ and/or Nrp1^−^ pTregs in colonic Treg compartment (107, 114). Also, the frequency of colonic Foxp3^+^Helios^−^Nrp1^−^ Tregs is significantly reduced in GF mice compared to SPF mice (116). Furthermore, treatment of SPF mice with broad-spectrum antibiotics decreased the Helios^−^ colonic Tregs (111). Molecular studies on *Foxp3* gene locus have identified an enhancer, CNS1, to have a prominent role in pTreg generation in gut-associated lymphoid tissues (46). CNS1 contains a TGFβ-NFAT response element (46) and binding sites for retinoic acid receptor (RAR) and retinoid X receptor heterodimer, receptor for retinoic acid (RA) (120). It has been shown that TGFβ and RA can induce *de novo* generation of pTregs upon antigen activation *via* CD103^+^ DCs (121, 122) (Figure 3). Indeed, CNS1-deficient mice have fewer LP Tregs at weaning (46). However, it was recently reported that CNS1 deficiency in colonic TCR transgenic T-cells only delays the pTreg generation and ultimately T-cells do convert to Foxp3^+^ Tregs (114).

While pTregs are largely accepted to be the primary source of intestinal Treg population, at least under an experimental setting where generation of extra-thymic Tregs are compromised, thymically generated Tregs can migrate to intestinal LP and proliferate to fill up the niche (Figure 3). It has been reported that in a limited TCR model, the TCR repertoire of tTregs and colonic Tregs overlap considerably (123). Another study utilizing K14-Aβb (Keratin 14 transgenic, K14) mice, which have restricted MHCII expression in thymic cortical epithelium and thus, cannot provide peripheral MHCII signals for extra-thymic Treg generation, showed that while Treg population was significantly decreased in mLNs and spleen, the intestinal Treg compartment was rather intact (124). The LP Treg niche of these mice was found to be inhabited by Tregs of thymic origin at young age. This niche filling phenomenon was not IL2 dependent but was induced by microbial presence, as broad-spectrum antibiotic treatment decreased both large and siLP-Tregs (siTregs) (Figure 3). At an older age however; newly generated tTregs were excluded from the LP, presumably due to already occupied Treg niche (124). Taken together, therefore, the available data suggest a peripheral origin of majority of intestinal Tregs and thymic origin of a small subset. However, these mechanisms of origin and development cannot be mutually exclusive and contributions from different pathways are likely to fine tune the ultimate composition of the intestinal Treg compartment.

### Tregs Specialize into Multiple Subsets in Intestines

**Figure 4:**
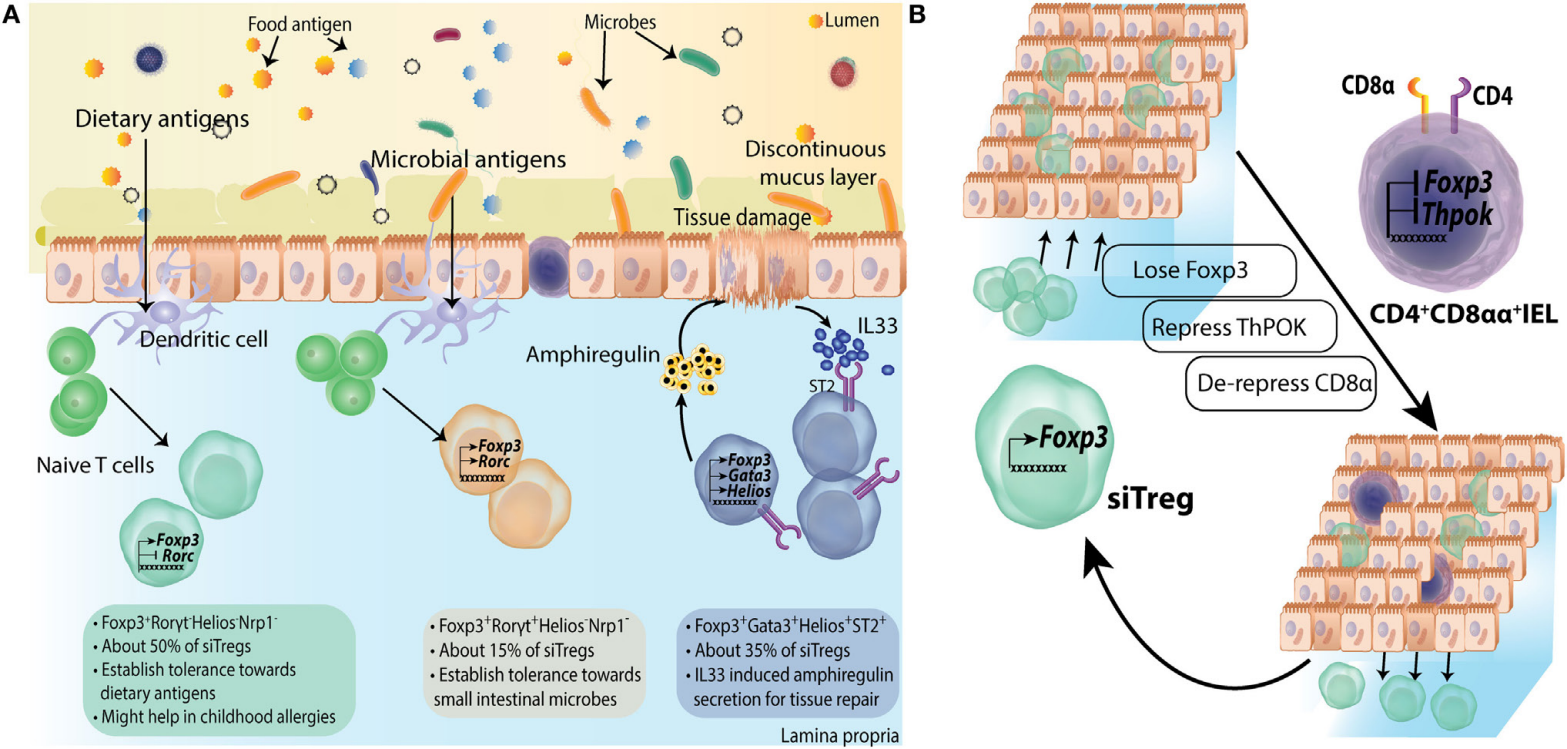
Subtype differentiation of regulatory T-cells (Tregs) in small intestine. **(A)** Small intestine lamina propria (LP) Tregs are differentiated into all three subtypes mentioned in Figure 3; however, Foxp3^+^RORγt^−^ Tregs are primary population forming about 50% of siTreg compartment. They are primarily generated in response to dietary macromolecules and are proposed to be helpful in containing childhood allergies. Foxp3^+^RORγt^+^ and Foxp3^+^GATA3^+^ Tregs are about 15% and 35% of total siTregs, respectively. **(B)** Some siTregs continuously traverse in and out of small intestinal epithelial compartment. While most of these Tregs return back to LP, a fraction of these lose their Foxp3 expression and repress ThPOK expression inside epithelial layer. They subsequently, de-repress *CD8a* locus to convert into CD4^+^CD8αα^+^ double positive intra epithelial lymphocytes.

Transcriptomic and functional analyses of intestinal Tregs have largely identified three specialized subsets. Based on transcription factor and surface molecular expression these subsets are GATA3^+^Helios^+^(Nrp1^+^), retinoic acid receptor related orphan receptor γt (RORγt) expressing RORγt^+^Helios^−^ and RORγt^−^Nrp1^−^(Helios^−^) subsets (Figures 3 and 4A).

GATA3 is expressed by about a third of intestinal Tregs and can be induced in CD4^+^Foxp3^+^ Tregs upon TCR engagement (110). That most of these Tregs are Helios^+^ and are unaffected by microbial presence suggests their thymic origin (107). However, upon TCR engagement, GATA3 can be induced in CD4^+^Foxp3^−^ naïve T-cells as well, in both *in vivo* and Treg polarizing *in vitro* settings (110). While expression of GATA3 is not required under homeostatic conditions, under inflammatory conditions lack of GATA3 in Tregs hampers their accumulation at inflammatory sites (110). GATA3 and Foxp3 interact both at protein and gene levels in Tregs (47). GATA3 binds to *Foxp3* locus and its deletion in Tregs reduces Foxp3 expression (47, 125). It occupies significant number of genes, which are direct targets of Foxp3 and thus, collaborates with Foxp3 to establish Treg gene expression program. Indeed, Treg-specific GATA3 deletion results in intestinal pathology with heightened Th2 cytokine production from large intestinal effector T-cells (47). Most of the GATA3^+^ Tregs in colon express ST2, which is regulated by GATA3 expression in a feed-forward manner (108). Alarmins like IL33 are produced upon local tissue damage (126, 127) and thus limit self-injury in part by activating GATA3^+^ST2^+^ Tregs (108). ST2^+^ Tregs show enhanced production and activation of IL10 and TGFβ (128). IL23 on the other hand appears to regulate the effect of IL33 in thymus-derived Tregs *via* STAT3 signaling (108).

CD4^+^Foxp3^+^RORγt^+^ Tregs in the intestine are another subset of Tregs specialized toward microbial immunity. These comprise about 50% of total Tregs in colon, which are readily lost in GF mice or upon antibiotic treatment (107, 129) (Figure 3). A small population consisting of about 10–15% of Tregs is also found in small intestine (107) (Figure 4). Most of the RORγt^+^ Tregs do not express Helios (107, 129) or Nrp1 (130), suggestive of their extra-thymic origin. However, these cells have substantially reduced CpG methylation within the CNS2 enhancer region of *Foxp3* locus, which is known to be well correlated with stable Foxp3 expression (130, 131). Not all bacteria can elicit similar population of RORγt^+^ Tregs, as it was found that a gradation of Treg inducing capacity exists (107). Mechanistic insights on why certain bacteria have superior capacity of inducing intestinal Tregs than others have started to emerge only recently. It has been reported that short chain fatty acids produced upon fermentation of starch and other dietary fibers by clostridia strains, mainly butyrate and propionate but not acetate, can contribute to colonic Treg generation. Mechanistically, this is attributed to their histone deacetylase inhibitory properties (132) resulting in increased acetylation of *Foxp3* locus (133, 134) (Figure 3). Apart from directly acting on T-cells, butyrate also affects DC ability to induce Treg differentiation. Knockdown of *Relb*, which encodes NFκB subunit, has been shown to generate tolerogenic DCs by inhibiting their maturation (135, 136). Indeed, *in vitro* treatment with butyrate represses lipopolysachharide response genes, including *Il12, Il6*, and *Relb* in DCs (133) (Figure 3). On a translational note, in human IBD patients, colonic butyrate producing bacteria are decreased (137) and mucosal butyrate transporter, monocarboxylate transporter 1, is downregulated (138). It is also possible that colonic Tregs are generated in an antigen-specific manner. Indeed, colonic Treg TCRs have been reported to interact with colonic bacteria *in vitro* (113). Very recently, colonic T cells with TCRs cognate to epitopes of a pathobiont *Helicobacter hepaticus* are shown to induce pTregs under homeostatic conditions (139). This study establishes the role of colonic pTregs in induction and maintenance of tolerance to pathobionts as well. Surprisingly, it was found that although such Tregs are RORγt^+^, their major functional suppressive capabilities are implemented by expression of the transcription factor cMAF (139) (Figure 3). cMAF offsets Th17 polarization by producing IL10 downstream to TGFβ1-STAT3 signaling (140). *H. hepaticus* colonized mice with Treg specific *Rorc* deletion had no significant increase in colonic Th17 cells while mice with Treg specific *Maf* deletion had significantly high Th17 cells (139). Thus, RORγt^+^ pTregs in colon establish tolerance to commensals as well as pathobionts and suppress inflammation in a cMAF-dependent manner.

The third Treg subtype (CD4^+^Foxp3^+^RORγt^−^) was identified very recently. Most of these cells express low levels of Nrp1 and thus are, supposedly, pTregs. These cells constitute about 50% of siLP Tregs and 15% of colonic Tregs (109) (Figures 3 and 4A). Their localization suggests that these are primarily generated against dietary antigens. Indeed, long-term antibiotic treatment of SPF mice could not reduce their numbers in intestine while RORγt^+^Nrp1^lo^ pTregs were reduced several folds. On the other hand, weaning SPF mice onto AF diet dramatically reduced RORγt^−^Nrp1^−^ pTregs (109). Adoptive transfer of naïve OTII CD4^+^ T-cells in mice on AF diet primarily elicits Th1 cell immune response while Th17 and Th2 responses were comparable to SPF animals. However, pTregs generated in this model were primarily RORγt^+^Nrp1^−^ pTregs, suggesting that dietary antigens can also generate RORγt^+^ pTregs in the absence of microbiota (109).

While TGFβ is by far the most important factor as far as generation and intestinal accumulation of pTregs is concerned (141–143), a generalized modulation of intestinal pTregs can be achieved by several other factors like dietary vitamin A, vitamin D, Niacin (Vitamin B3), Folic acid (Vitamin B9), and tryptophan [reviewed in Ref. (144)]. All-trans retinoic acid, a metabolite of Vitamin A, produced by DCs facilitates *de novo* generation of Foxp3^+^ Tregs from naive CD4^+^CD25^−^ T-cell populations in mice (121, 145) (Figure 3). It also plays an important role in upregulating gut-homing markers CCR9 and CD103 (integrin αE) on pTregs (146). Feeding mice with Vitamin A-deficient diet or treatment with RAR inhibitors reduces the RORγt^+^ pTregs in colon while GATA3^+^ Tregs are not affected (129). Similarly, RORγt^−^ pTregs in small intestine are also not affected by vitamin A or RA (109). Vitamin D metabolite 1, 25-dihyroxyvitamin D3 binds to Vitamin D3 nuclear receptor in CD4^+^ T-cells and promotes *Foxp3* expression (147) (Figure 3). Recently, it was shown that in human patients of ulcerative colitis a Vitamin D3 agonist can convert CD4^+^ T-cells to pTregs (148).

### Functions of Intestinal Tregs

Foxp3^+^GATA3^+^ intestinal Tregs express high level of ST2 (108). These Tregs express high levels of tissue repair factor, an EGF like growth factor Amphiregulin (108, 149) (Figures 3 and 4A). It appears that Amphiregulin mediated tissue repair might be a generalized mechanism employed by tissue-resident Tregs as exemplified by its evolving role in lung resident as well as intratumoral Tregs (150, 151). Foxp3^+^RORγt^+^ Tregs express increased levels of ICOS, CTLA4, and the nucleotidases CD39 and CD73 altogether, indicating robust regulatory functions (107, 129). Interestingly, Foxp3^+^RORγt^+^ Tregs have been implicated in regulating both Th2 and Th1/Th17 mediated immunity in two independent studies, implicating animal housing conditions as an important determinant of the type of immune response (107, 129). siLP Foxp3^+^RORγt^−^ Tregs primarily work toward containment of Th1 immunity. When OTII T-cells are transferred in AF mice, T-cells primarily convert to IFNγ^+^ OTII cells and induced Tregs are mainly Tbet^+^, although the extent of such RORγt^−^ pTreg induction in AF conditions was severely compromised compared to SPF conditions (109). As a consequence, in an experimental model of food allergy, where SPF BALB/c mice were weaned onto an amino acid diet, higher diarrheal instances were reported than mice on normal chow (109). While these results underscore an important function of RORγt^−^ pTregs in curbing food allergy, further transcriptomic and phenotypic analyses might provide additional clues to their functions.

Of note, one more function of siTregs was identified recently while looking at the IEL population. It was observed that LP Tregs migrate to epithelial compartment as well, where a fraction of them lose Foxp3 expression (152). Further, these Tregs then give up ThPOK expression leading to de-repression of *Cd8* locus and thus, get converted into a CD4^+^CD8αα^+^ IELs (152) (Figure 4B). IELs have cytotoxic as well as immunoregulatory machinery suggesting a role in both mucosal barrier maintenance and elimination of stressed intestinal epithelial cells (153).

## Skin Tregs: Keeps Your Hair on

Skin is the largest organ amounting to almost 15% of adult human body weight. Being our exterior, it is always exposed to environmental, microbial, physical, and chemical insults. Skin also harbors more than 10^12^ bacteria/m^2^ (154) in the surface intercorneocytic spaces.

### Skin Architecture

Anatomically, skin is composed of three layers, the outer epidermis, middle dermis, and inner subcutaneous tissue layer (155). The terminally differentiated keratinocytes in epidermis synthesize long, thread-like protective protein keratin and form a physical barrier. Products of various sweat and sebaceous glands interspersed at epidermal–dermal junction along with antimicrobial peptides develop an acidic hydrophilic skin which acts as a biochemical barrier (154). The epidermal–dermal junction also hosts hair follicles. Cellular component of epidermis comprises of Langerhans cells (specialized skin DCs) and T lymphocytes. The dermis is composed of layers of thick and thin collagen fibers which provide mechanical framework to host blood vessels and various immune cells like dermal DCs, αβ and γδ T-cells, NK cells, B cells, macrophages, and mast cells (154). Understandably, given the exposed nature of skin, it is highly vulnerable to overzealous immune responses against skin commensals and self-antigens. Tregs are an important component of establishing tolerance and homeostasis in the skin. Indeed, both scurfy mouse and human IPEX patients present fulminating immune responses in skin. A study, examining children with IPEX syndrome reported that more than 70% children presented Atopic Dermatitis and eczema with 1.5 months as median age of onset of symptoms (156).

### Skin Treg Origin and Accumulation

Normally, 30–50% and 20–30% of total CD4^+^ T-cells are Tregs in mouse and human skin, respectively (157, 158). It is difficult to establish the origin of cutaneous Tregs, given the paucity of specific information. However, a wave of Tregs has been shown to populate the skin in early neonatal period in a skin bacterial colonization model in mice (159). Furthermore, restricting lymphoid emigration of T-cells by treating with sphingosine-1-phosphate receptor antagonist FTY720 resulted in preferential accumulation of Tregs in thymus instead of skin draining LNs, suggesting their migration directly from thymus (159). In humans, the cutaneous Tregs and Tconv cells share very few TCRβ sequences and these Tregs present a fully demethylated *FOXP3* CNS2 region, suggestive of their stability and thymic origin (157, 160, 161). This is little surprising as skin Tregs establish tolerance to not only self-antigens but also to commensals. How are then commensal antigen-specific Tregs generated in thymus? One possibility appears to be plasmacytoid DCs (pDCs) that have been shown to be able to take up innocuous peripheral antigens to thymus (162) and LNs (163) to induce tolerance. Generally, pDCs are not present in skin but can accumulate in the presence of inflammatory conditions (164, 165). Another probability is that some tTregs have TCRs with sufficient cross-reactivity to microbial antigens and thus can specifically expand and accumulate at sites where antigen is present. However, if indeed skin Tregs are thymic by origin, the concerning mechanisms remain to be elucidated.

Modeling of inducible expression of a self-antigen, by fusing transferrin receptor transmembrane domain, GFP and amino acids 230–359 of chicken ovalbumin in mouse epidermis, revealed that circulating Tregs are not able to suppress primary immune response against OVA, though it initiated activation of Tregs (166). The inflammation resolved spontaneously and any subsequent antigen expression led to an attenuated and short immune response. Further analysis revealed that a fraction of Tregs persisted in the skin which expressed low level of CD25 but higher KLRG1, CTLA4, and CD127, akin to the memory T-cells (166). It is to be noted here that a recent study examining the transcriptional, epigenomic, and functional changes in inflammation experienced Tregs employing the Foxp3^DTR^ system, presented that Tregs revert most of the activation induced changes and lose the accentuated suppressive ability over time (167). An earlier report revealed that cutaneous Tregs express CCR4 and adhesion molecule Integrin αE, CD103 (168). In a mixed bone marrow chimera study, CCR4-deficient Tregs could not reconstitute the skin Treg compartment (168). CCL17 and CCL22 are known chemokine ligands for CCR4 (169, 170), which are differentially expressed in inflamed skin mainly by endothelial cells and dermal DCs, respectively (171). These molecules sequentially manage T-cell homing to skin, where CCL17 promotes vascular recognition and extravasation and CCL22 guides subsequent migration in skin (172). More than 70% skin Tregs express GATA3, although its deletion in Tregs does not alter Treg profile or cause overt skin related phenotype under homeostatic conditions (110).

**Figure 5:**
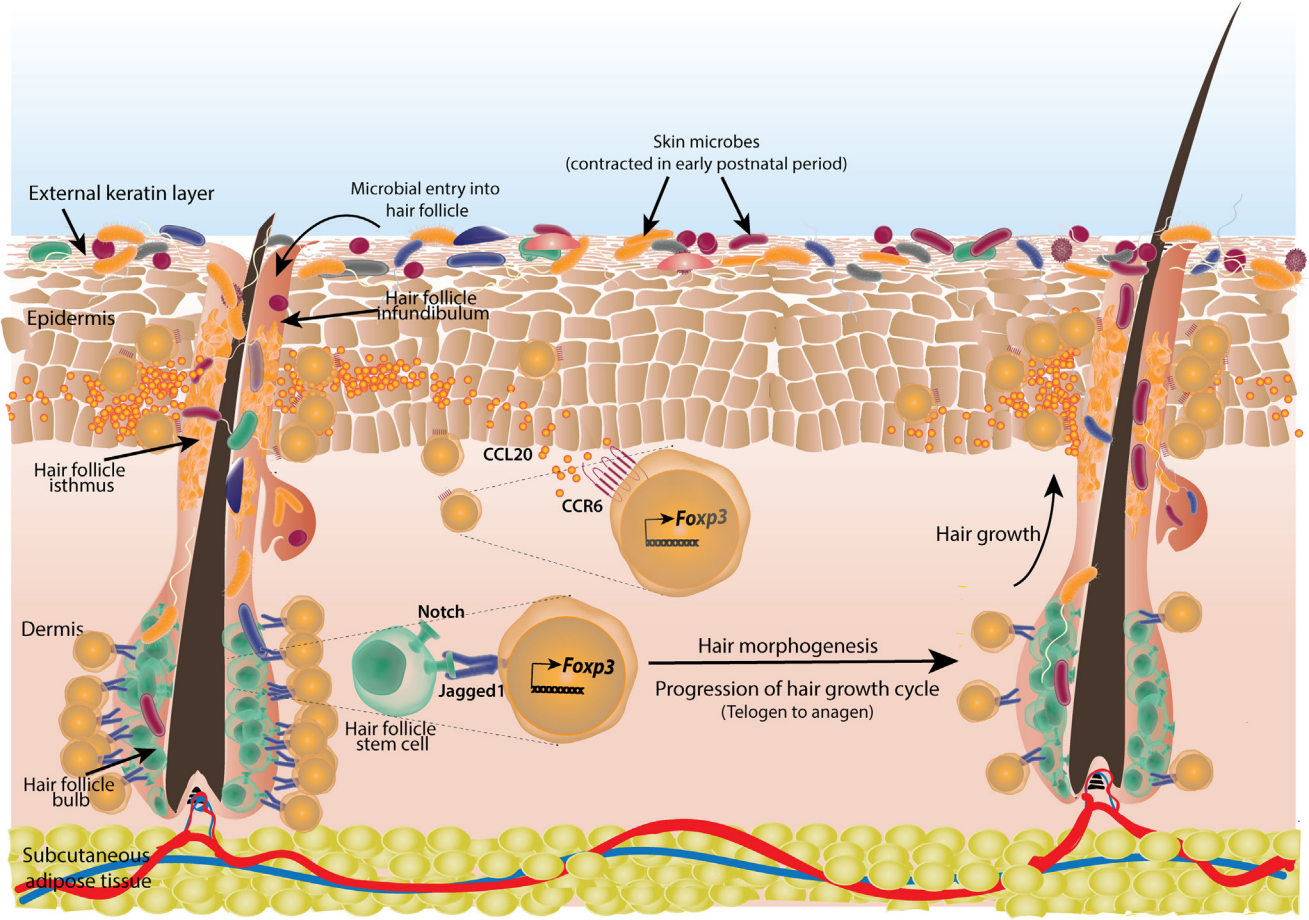
Cutaneous regulatory T-cells (Tregs) facilitate hair morphogenesis and establish tolerance to skin commensals. Skin Tregs colonize mouse skin in early postnatal period coinciding with initial microbial colonization and hair follicle development. Skin microbes enter the hair follicle which induces production of chemokine CCL20 from infundibular cells that attracts large number of Tregs expressing corresponding receptor CCR6. The accumulated Tregs subsequently establish tolerance to these commensals. Also, during the resting phase of hair growth (telogen) which initially coincides with commensal entry in hair follicle, large number of Tregs are seen in contact with hair follicle stem cells (HFSCs) in the bulb of follicle. These Tregs provide notch ligand Jagged1 to the stem cells. Notch signaling triggers the active phase of hair growth cycle (anagen). Tregs are reduced in anagen phase around HFSCs.

Since skin is heavily exposed to commensals as well as pathogens, it is imperative to speculate that a fine tuning of effector and suppressive immune responses has evolved. The commensal microbiota residing within skin has been shown to calibrate barrier immunity (173, 174). To identify mechanisms behind development of tolerance to skin commensals, Scharschmidt et al. performed some elegant experiments with model peptide antigen expressing *Staphylococcous epidermidis*, a human skin commensal which efficiently colonizes mouse skin (159). Surprisingly, skin colonization in adult mice did not evoke any tolerance to bacteria, as seen by inflammatory response upon re-challenge. However, when neonatal mice were colonized for a week during postnatal week 2, there was an appreciable attenuation of inflammatory response upon re-challenge after 3–4 weeks. Subdued response was associated with marked enrichment of antigen-specific Tregs in skin and draining LNs and the tolerance could be reversed upon FTY720 treatment (159). Another study has also shown that Tregs, generated very early in life in a defined perinatal window, play a very distinct role in maintaining self-tolerance (175). Thus, in mice, an abrupt wave of Treg infiltration occurs in a defined early postnatal period to establish dominant tolerance toward skin commensals (159) (Figure 5).

Both Tregs and skin commensals are localized to hair follicles and in mouse, the time of Treg infiltration (week 2 postnatal) is coincident with hair follicle development (176, 177). Thus, it was speculated that hair follicles might have a role in ingress of Tregs in skin. It has been shown that chemokines from different parts of hair follicles like CCL2 from isthmus, CCL20 from infundibulum, and CCL8 from bulge keratinocytes generate specific type of Langerhans cells (178). Similarly, in a mouse model of skin specific hair follicle morphogenesis arrest, skin Treg population was reduced without affecting Treg population in intestine or draining LNs in neonatal mice (179). Further investigations revealed that neonatal SPF mouse skin produces high amount of chemokine CCL20 whose receptor CCR6 was found to be enriched on cutaneous Tregs (Figure 5). It was confirmed by adoptive transfer experiments that CCR6-deficient Tregs were at a competitive disadvantage to reconstitute skin T-cell compartment (179). Tregs in neonates also express high CCR8 and its ligand CCL22 is, in turn, expressed in skin. However, their contribution in establishing and/or maintenance of skin Tregs is yet to be examined. CCL20 mRNA expression also increases in human fetal skin explants upon exposure to cutaneous commensals and bacterial components (179). Thus, tissue morphogenesis (hair follicle generation and subsequent chemokine production by the cells of hair follicle) and commensals are shown to cooperate in developing a tissue-specific immune tolerance. It would be interesting to extrapolate this model to other microbe inhabited organ systems. Whether, these mechanisms sustain throughout the life span particularly in adult life is not known. Earlier, 8- to 12-week-old GF mice were reported to have about twofold more cutaneous Tregs than SPF animals (180). It is possible that yet unknown factors other than commensals increase Treg population in adult GF skin or a lack of tuned immune response against commensals diverts immune resources toward self-antigens and in turn, progressively enrich self-antigen-specific Tregs in skin.

**Figure 6:**
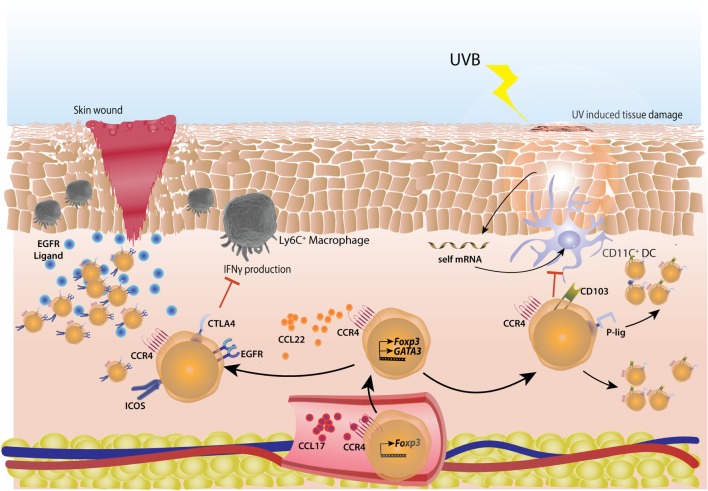
Cutaneous regulatory T-cells (Tregs) actively repair skin tissue damage. Tregs migrate to skin by virtue of sequential interactions of chemokines CCL17 and CCL22 with their receptor CCR4. CCL17 is secreted by endothelial cells in inflamed skin and helps in extravasation of Tregs, CCL22 manages subsequent migration of Tregs. In case of skin wound-induced inflammation, Tregs acquire a highly activated phenotype with higher surface expression of CTLA4 and ICOS. These Tregs actively suppress IFNγ production from inflammatory Ly6C^+^ macrophages. Also, Tregs express high amount of EGFR, helping in their tissue repair ability. In case of short wave UVB light induced skin damage, self mRNA from keratinocytes are taken up by CD103^+^ dendritic cells (DCs) which induce the cutaneous inflammation. These DCs are suppressed by Tregs which have high surface expression of CD103 and P-lig and thus can migrate into non-inflamed skin as well, subsequent to UV exposure.

Short wavelength Ultraviolet (UVB) exposure also enriches and accumulates Tregs in mouse skin (181). UVB exposure damages self-RNA in keratinocytes which is sensed by TLR3 to generate an inflammatory response (182). Indeed, the Tregs accumulating post-UVB exposure were Nrp1^+^ with highly demethylated *Foxp3* CNS2 (181). These Tregs highly expressed homing molecules like CD103, CCR4, and P-lig and thus, were able to migrate to the non-UVB exposed parts of the skin as well (181) (Figure 6). Interestingly, UVB phototherapy is an effective treatment in autoimmune skin conditions, like psoriasis (183) and atopic dermatitis (184).

### Tissue Adaptations and Homeostatic Functions

True to the nature of tissue-resident Tregs where they have been shown to not only develop immune tolerance but also get accustomed to and help local physiology, cutaneous Tregs in hair follicles have been found to modulate hair follicle stem cells (HFSCs) in addition to establishing tolerance to commensals. In mouse skin, an abundance of Tregs was found during telogen phase of hair follicle growth which is characterized by quiescence only to be followed by an active proliferation phase called anagen. Treg population decreases during anagen phase. And as these phases alternate in mouse skin so are the numbers of hair follicle associated Tregs (158) (Figure 5). Animals with transient depletion of Tregs during telogen, failed to progress to anagen phase and could not re-grow hairs (158). It was not inflammation related, as transient depletion of Tregs did not elicit any major inflammatory event in skin and co-depletion of either effector CD4^+^, CD8^+^, Gr-1 expressing neutrophils or CD11C^+^ myeloid cells did not rescue HFSC proliferation (158). This suggested a non-immune role of Tregs in HFSC proliferation. Akin to Tregs in hematopoietic system which form an immune-privileged site to provide a protective niche to hematopoietic stem cells (185), hair follicle Tregs were found to co-localize with HFSCs. These Tregs highly express “*jagged1*” which encodes a ligand for notch signaling pathway responsible for HFSC proliferation (158) (Figure 5).

Regulatory T-cells play an important role in cutaneous wound healing as well. They accumulate in large numbers at the site soon after a wound injury in skin (186). These Tregs are of activated phenotype with high CD25, CTLA4, and ICOS expression and limit the IFNγ producing T-cells and inflammatory macrophages in wounds (186). The Tregs involved in cutaneous wound healing were shown to be dependent on EGFR pathway (186) as in lung and muscle-healing Tregs (150, 187, 188) (Figure 6).

## Tumor-Infiltrating Regulatory T-Cells (TI-Tregs): A Battle Won, War Lost

Tumors are wounds that do not heal (189–191). Solid tumors, in particular, heterogeneously indulge in various stages of wound healing, which provide essential growth factors for the tumor growth. This hijack of natural processes results in heightened inflammation and its subsequent resolution in the tumor microenvironment, presumably setting up a vicious cycle. Inflammation on one hand provides growth and metastasis opportunities; resolution of inflammation helps the tumor to escape antitumor immunity. Therefore, it is not surprising that many solid tumors including hepatocellular (192), gastric (193), lung (194), breast (195), ovarian (196), cervical (197), and melanomas (198) summon comparatively large numbers of Tregs which sometimes account for even more than 50% of CD4^+^ T-cell compartment (199). For most of the tumors, the presence of high number of Tregs indicates a guarded to grave prognosis. However, several studies reported a favorable role of FOXP3^+^ T-cells in colorectal carcinomas (CRC) (200–202). It is to be noted that FOXP3 expression is not exclusive to *bona fide* Tregs in humans, often, effector T-cells express FOXP3, albeit transiently (203, 204). Based on expression levels of FOXP3 and Protein tyrosine phosphatase isoform A (CD45RA), human peripheral blood FOXP3^+^CD4^+^ T-cells can be classified into FOXP3^hi^CD45RA^−^ *bona fide* Tregs which are highly suppressive and phenotypically eTregs; FOXP3^lo^CD45RA^+^ naïve T-cells and FOXP3^lo^CD45RA^−^ effector T-cells which are not suppressive in an *in vitro* suppression assay (203). Indeed, careful analysis of TILs in human CRC by Saito et al. (205) revealed the heterogeneity of FOXP3 expression. The authors identified that CRC, where higher expression of FOXP3 was associated with favorable outcomes, were actually infiltrated more with FOXP3^lo^CD45RA^+^ effector T-cells and upregulated inflammatory genes like *Il12a, Il12b, Tgfb1*, and *Tnf*. Higher infiltration of FOXP3^hi^CD45RA^−^ cells resulted in poor prognosis and lower disease-free survival (205) as reported for other tumors.

### Origin and Accumulation of TI-Tregs

**Figure 7:**
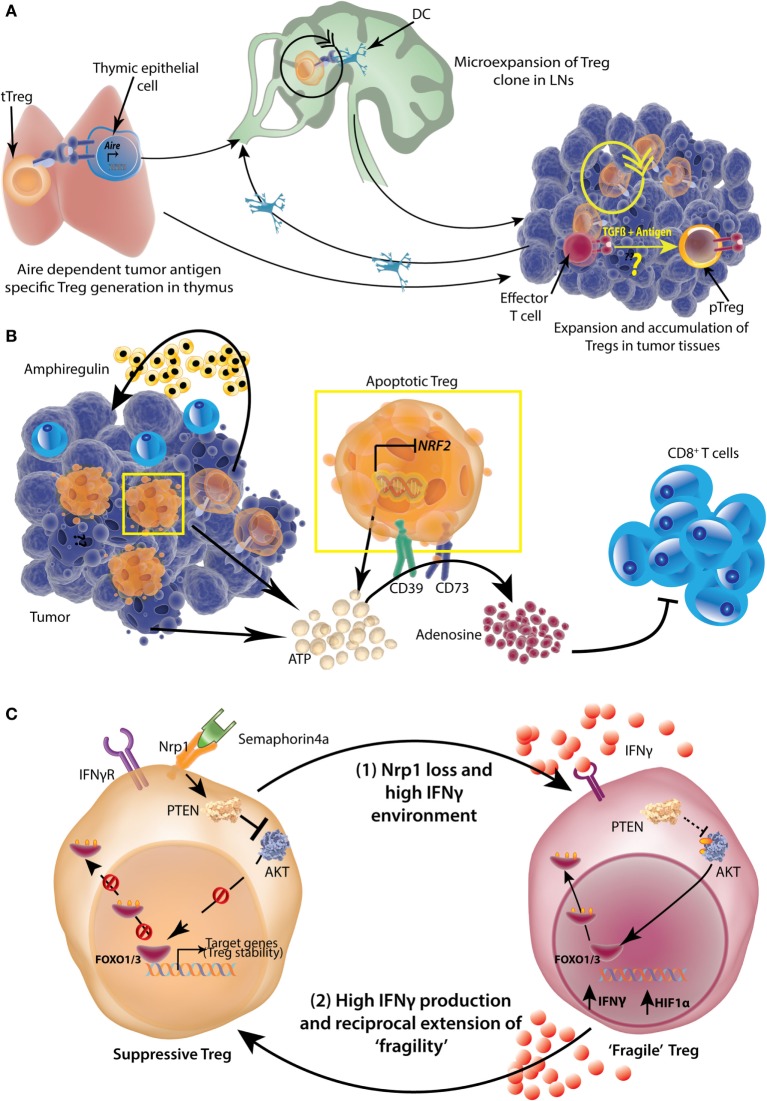
Origin, accumulation, and functional potentiation of tumor-infiltrating regulatory T-cells (Tregs). **(A)** Tumor-specific antigens can be expressed by thymic epithelial cells in an *Aire*-dependent manner, which then select the tumor antigen-specific Tregs. These Tregs then expand in tumor draining lymph nodes (LNs) with the help of dendritic cells (DCs). Tregs can also be directly recruited to tumors and undergo expansion there. While intratumoral conversion of effector T-cells to pTregs is likely, the extent to which this occurs under physiological conditions is not completely understood. **(B)** A high number of TI-Tregs are apoptotic because of suppressed expression of *Nrf2*. These Tregs as well as dying tumor cells release copious amounts of ATP, which is converted to adenosine by Treg ectoenzymes CD39 and CD73 in a sequential manner. The resulting adenosine is highly suppressive to tumor-infiltrating CD8^+^ effector T-cells. Further, Tregs also produce Amphiregulin in certain tumor types, which help in tumor progression. **(C)** More than 50% of TI-Tregs express surface Neuropilin1 (Nrp1), which is a receptor for Semaphorin 4a. Upon ligation with Semaphorin4a, Nrp1 activates lipid phosphatase Phosphatase and tensin homolog (PTEN) which promptly dephosphorylates AKT rendering its sequestration in cytosol and nuclear retention of Foxo1/3 transcription factors which help in stabilization and survival of Tregs. A loss of Nrp1 renders Treg highly susceptible to IFNγ and such Tregs also produce high amount of IFNγ and HIF1α (“Fragile” Tregs). The resultant high IFNγ environment, reciprocally, can induce “fragility” in other Nrp1-sufficient Tregs as well, setting up a vicious cycle.

As tTregs and pTregs differ in their stability, conclusive information about origin of TI-Tregs can be very valuable to design TI-Treg specific therapies in cancers. Tumors drive immune responses against tumor-associated self-antigens as well as tumor-specific neo-self antigens. Thus, in theory, Tregs against self-antigens (tTregs) and pTregs against neo-self antigens are possible. Presence of high levels of TGFβ in most solid tumors reinforces the idea of generation of pTregs in tumor microenvironments (206, 207). However, TI-Tregs in several murine tumors have been shown to express high levels of Nrp1 and Helios proteins, suggestive of a thymic origin (208). Attempts for *in situ* conversion of conventional CD4^+^ T-cells to Tregs against tumors expressing model antigens did result in intratumoral pTregs generation (209, 210), but monoclonal populations of antigen-specific T-cells do not recapitulate physiological conditions where antigen-specific T-cells represent less than 5% of TILs (211–213). TCR repertoire analyses have revealed almost no overlap in Foxp3^+^ and Foxp3^−^ T-cells in autochthonous prostate tumors (214), carcinogen-induced tumors (215), and in heterotopic transplanted tumors in mice (216). Further, these studies confirmed that there is enrichment and expansion of selective TCR bearing Tregs inside tumor microenvironment (214, 216). In human breast cancers (217) and hepatitis B virus positive hepatocellular carcinomas (HCC) (192), very low TCR repertoire overlap between TI-Tregs and conventional T-cells suggests little to no conversion of conventional CD4^+^ T-cells into Tregs (Figure 7A). Malchow et al. (214) developed a transgenic mouse expressing the model oncogene SV40 T antigen in prostate and a fixed TCRβ chain (TRAMP-Foxp3^eGFP^TCRβ^Tg^). They found that Tregs expressing a single TCR (MJ23), reactive to a normally expressed prostate antigen, consistently populated the tumors (214). This TCR was able to drive a tTreg clone development. However, a deficiency of the transcription factor autoimmune regulator abolished development of these clones (214), suggesting that at least in these experimental settings, TI-Tregs are generated in thymus against a normal tissue expressed self-antigen (Figure 7A). Recently, two MHCII restricted natural self-antigen ligands of MJ23 Tregs were discovered. Both of these ligands are non-overlapping peptides originating from same prostatic protein (Tcaf3) and while one is expressed in mouse prostate tumors (MJ23), the other is associated with prostatic autoimmune lesions (SP33) (218). Another study focusing on epigenetic hallmarks of tTregs found that TI-Tregs had consistently hypomethylated *Foxp3* CNS2 in various orthotopic and heterotopic transplanted tumor models, even at different time points of tumor growth (219). These findings were further confirmed in TI-Tregs from different human tumors. It is to be noted that there have been equivocal reports about the demethylated CNS2 being specific for tTregs, since *Foxp3* CNS2 region in pTregs has also been shown to be demethylated (220, 221), most likely upon eventual stabilization following its *de novo* induction.

Overall, available evidences largely point toward higher enrichment and expansion of tTregs inside solid tumors. However, why should not higher TGFβ levels and an activating tumor environment drive pTreg generation and expansion is a baffling question. Probably more conclusive lineage tracing experiments across tumors can be proven more insightful.

### TI-Tregs Add Another Layer of Diversification

Recent studies analyzing transcriptome of TI-Tregs from human cancers have identified that though TI-Tregs are largely similar to normal tissue-resident Tregs, these have some specific characteristics and molecular patterns which can be utilized for selective therapy (192, 217, 222). Plitas et al. (217) found that breast cancers, with rather aggressive phenotype, were enriched for Tregs, which were highly suppressive in a microsuppression assay and were highly proliferative based on increased expression of nuclear protein Ki67, a cellular marker for proliferation (223). Further transcriptome analysis of TI-Tregs from breast cancers and gastric cancers as well as brain metastases of NSCLC and liver metastasis of CRC, suggested a signature gene set for these cells (222). TI-Tregs expressed BATF, CCR8, CD30, IL1R2, IL21R, PDL1, and PDL2 along with FOXP3 and IL2Ra at a very high level (222). Recently, BATF was shown to be involved in context dependent gene set expression in tissue Tregs (224). In the IPEX patients, a gain-of-function mutation in FOXP3 locus (A384T) results in expanded DNA binding specificities of FOXP3. Its altered binding to *BATF* locus repressed BATF expression leading to repressed GATA3, ST2, and CCR4 expression in Tregs (224). These genes are significant in converting cTregs to eTregs, therefore, their decreased expression led to a widespread tissue-specific autoimmunity (224).

Genome-wide transcriptome analyses identified *MAGEH1*, Melanoma antigen family H1 gene, encoding a putative E3 ubiquitin ligase potentially regulating TI-Treg survival and function; Chemokine (C-C motif) receptor 8 (CCR8), known receptor for chemokines CCL1 (225), CCL8 (226), CCL16 (227), and CCL18 (228) in humans; CD177, a glycosyl-phosphatidylinositol-linked cell surface glycoprotein that can bind platelet endothelial cell adhesion molecule-1 and is known for neutrophil transmigration and survival (229); and *LAYN*, encoding a novel c-type lectin surface receptor layilin, a proposed receptor for Hyaluronan (230). However, subsequently, layilin has been found highly expressed on tumor-infiltrating cytotoxic CD8^+^ T-cells as well, particularly those with an exhaustive (highly expressing *CTLA4, PDCD1*, and *HAVCR2*) phenotype (192). Enrichment of *LAYN, MAGEH1*, and *CCR8* in whole tumor samples correlated significantly with reduced 5-year survival rate of CRC and NSCLC patients (222). CCR8 expression was exclusively enriched on TI-Tregs, whereas CCR2, CCR4, and CCR5 expressions were found on other tumor-infiltrating and/or peripheral blood Tconv cells as well. Indeed, a CCR4 depleting antibody has been shown to deplete both Tregs and Tconv cells (231). Some Tregs in draining LNs also expressed high CCR8 which might be ones earmarked for tumor infiltration or Tregs in micrometastases inside LNs (217). As peripheral blood and/or LN Tregs do not express CCR8, its importance in recruitment of Tregs to tumors is not appreciated. It is possible that CCR8 is expressed to retain Tregs in tumors. Indeed, CCR8 ligands like CCL1 and CCL18 are highly transcribed in tumor-infiltrating myeloid cells (217). Whether, CCR8 expression is an indicator of highly suppressive TI-Tregs or it has further functional importance is not yet known. However, human Tregs exposed to CCR8 ligand CCL1 and not CCL8, CCL16, or CCL18 induce surface CCR8 expression *via* a STAT3-mediated pathway (232). Such cells, subsequently, upregulate their expression of FOXP3, CD39, Granzyme B, and IL10 and are functionally more suppressive in a microsuppression assay and in a mouse model of multiple sclerosis (232).

### Function of TI-Tregs

There is a repertoire of known and yet unknown mechanisms which Tregs utilize to suppress an immune response. TI-Tregs also use similar mechanisms which include production of immunosuppressive cytokines like IL10 and TGFβ (233, 234); sequestration of IL2 (235); direct cytolysis of target lymphocytes using granzyme B and perforin (236); contact based immunosuppression using surface inhibitory molecules like CTLA4 (237), PD1 (238, 239), LAG3 (240), TIM3 (241), and CD39/CD73-generated adenosine-mediated T-cell suppression *via* adenosine receptor 2A (242, 243). However, how the TI-Tregs have highly accentuated suppressive response is not very well understood. A large accumulation of Tregs might help in a collective exaggerated suppression, but it cannot explain individual potentiation. Recently, it was shown that TI-Tregs are highly apoptotic on account of comparatively low expression of the transcription factor Nuclear factor like 2 (NRF2) (244). NRF2 regulates antioxidant defense system in macrophages and epithelial cells (245). A lack of NRF2 makes TI-Tregs more apoptotic in high oxidative stress tumor microenvironment. But, owing to increased release of ATP and high CD73/CD39 expression, apoptotic TI-Tregs generate large amount of adenosine and thus, become even more suppressive (244) (Figure 7B). It is to be noted though that earlier Imatinib induced apoptosis of TI-Tregs was shown to enhance antitumor immunity (246).

TI-Tregs in human breast cancers (217) and HCC (192) highly express *Il1r2* gene encoding a decoy IL1 receptor. Also, TI-Tregs are found to be highly stable owing to the enhanced expression of lipid phosphatase Phosphatase and tensin homolog (PTEN) and VEGF receptor Nrp1 (199, 238, 247). Binding of Nrp1 to its ligand Semaphorin4a increased Foxo1 and Foxo3 nuclear localization by inhibiting AKT phosphorylation which stabilized Treg signature genes and antiapoptotic genes (247) (Figure 7C). AKT dephosphorylation was achieved by activation of PTEN by Nrp1. Indeed, mice with Treg specific PTEN deletion generate an accentuated antitumor immune response (238). Nrp1 expression is primarily important for TI-Tregs as the loss of Nrp1 even from a fraction of Tregs under appropriate experimental conditions, rendered all the Tregs (including those that were Nrp1 sufficient) “fragile” (199). This observation emphasizes that Tregs can not only modulate other immune cells but can phenotypically influence other Tregs as well. The TI-Treg fragility was shown to be induced by IFNγ production by Nrp1-deficient Tregs and exogenous IFNγ (199) (Figure 7C). The authors further show that HIF1α was a major factor induced in Nrp1-deficient and fragile Tregs, and both HIF1α and IFNγ can be induced by hypoxia (199). However, as most of the solid tumors become progressively hypoxic (248, 249), whether this phenomenon is prevalent in progressive tumors and if so, whether it is efficient for a significant regeneration of antitumor immune response, remains to be seen. TI-Tregs have been shown to highly express receptor activator of nuclear factor κB ligand (RANKL), which upon binding to its receptor RANK expressed in mammary carcinoma cells increases lung metastasis (250). RANKL has also been implicated in renewal of breast cancer progenitor cells (251) and metastasis of prostate cancers (252) by modulating protein kinase inhibitor of nuclear factor κB kinase α (IKKα). Overall, these findings suggest that there is a specific phenotypic and functional identity to TI-Tregs and thus, it is possible to selectively target them for triggering efficient antitumor immunity.

## Other Tregs in Tissue Inflammation and Homeostasis

As the diversity in characteristics and functions of tissue Tregs is being unraveled, several other interesting populations have been described which deserve more detailed phenotypic and functional characterization.

### Regeneration Powerhouse

**Figure 8:**
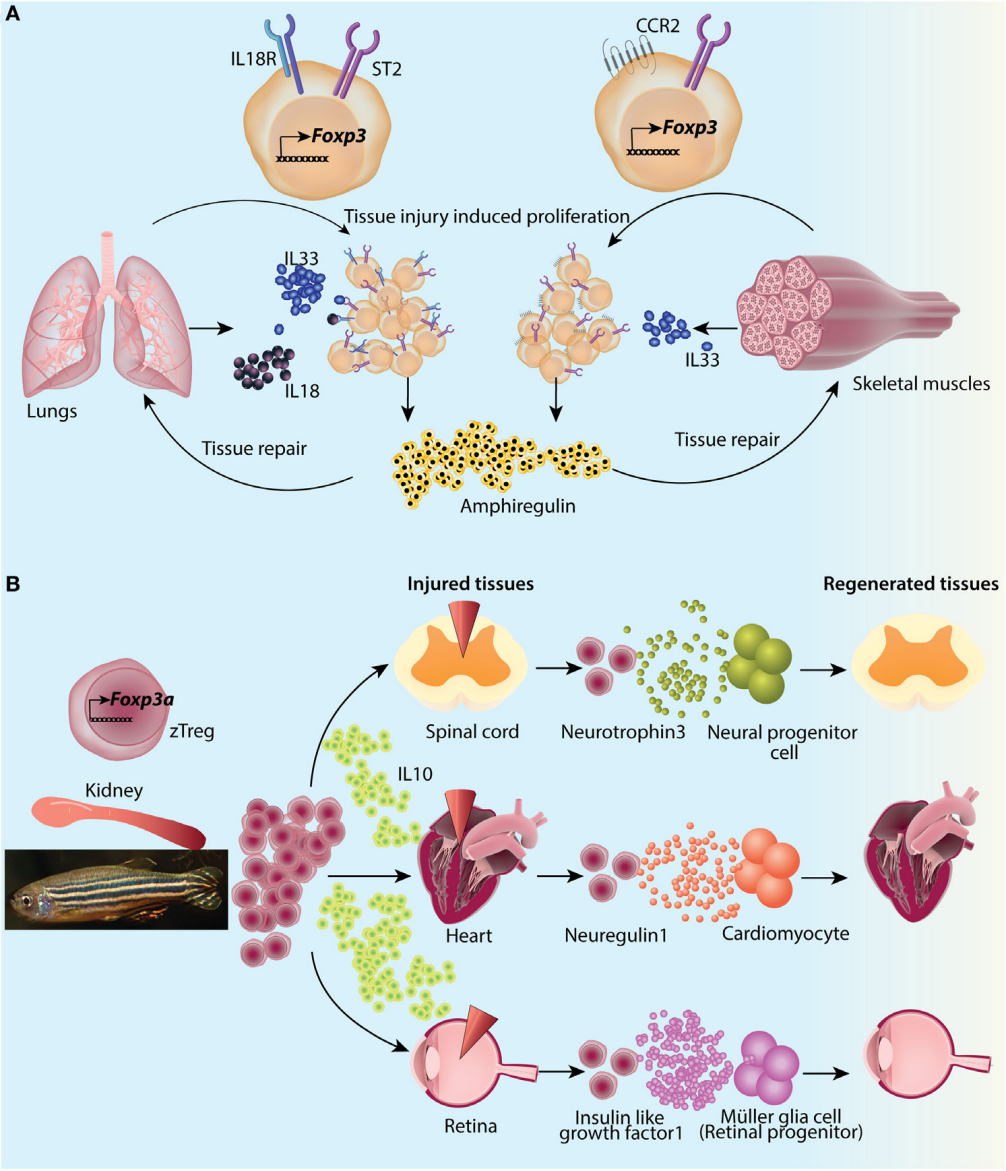
Emerging evidences highlight a compulsory requirement of regulatory T-cells (Tregs) in tissue regeneration and repair. **(A)** Both lungs and muscles contain population of Tregs which proliferate vigorously upon tissue injury. Lung reparative Tregs respond to both inflammatory IL18 and alarmin IL33 and produce amphiregulin in a TCR-independent manner. Muscle Tregs respond to IL33 produced upon muscle damage and produce amphiregulin for subsequent repair. **(B)** In zebrafish, mammalian *Foxp3* ortholog *Foxp3a* expressing Tregs (zTregs) are primarily present in kidney. However, upon tissue injury, they soon accumulate at the site of the injury. Apart from anti-inflammatory IL10 production, zTregs co-localize with organ progenitor cells and provide tissue-specific growth factors to progenitors like neurotrophin3 for neural progenitors in spinal cord, neuregulin1 for cardiomyocytes in heart, and insulin-like growth factor1 to Müller glial cells in retina.

It was reported earlier that in a non-inflammatory model of regenerative alveologenesis, Tregs enhanced epithelial proliferation. A Treg coculture with type II alveolar cells (AT2) increased their proliferation in CD103-dependent manner (253). In accordance to these findings, a distinct population of Tregs expressing high levels of pro-inflammatory cytokine IL18 receptor (IL18R) and ST2 has been described in lungs (150). IL18R^+^ Tregs expand early in the course of a lung injury and enhance tissue repair by producing a large amounts of tissue repair protein amphiregulin in an “innate” manner, independent of TCR engagement (150) (Figure 8A). In animals with Treg specific amphiregulin deficiency, a rapid decline in lung functions was observed upon intranasal influenza virus infection, while antiviral immune response was intact (150). Transcriptomic analysis revealed that these “repair Tregs” have a distinct gene expression pattern indicating their proficiency in extracellular matrix remodeling and tissue repair (150).

Another unique tissue Treg population has been found in skeletal muscles, where by virtue of amphiregulin secretion, they help in muscle regeneration and healing. These cells, usually accounting for 10% of muscle T-cells at a steady state, proliferate vigorously after an intramuscular administration of cardiotoxin, which induces hypercontraction and myofibril death induced acute injury (254), reaching close to 50% of muscle T-cell population (188). High expression of Nrp1 and Helios and a unique and restricted TCR repertoire suggests thymic origin of muscle Tregs and reactivity to a local muscle antigen (188). These Tregs have a unique transcriptome compared to lymphoid organ Tregs with several differentially expressed genes. They have upregulated transcripts involved in Treg mediated suppression (*Il10, Gzmb*, etc.), tissue repair (*Il1rl1, Areg*, etc.), and muscle regeneration (255) (*Ccr2*) as well as genes encoding proteins found in contractile muscle function (256) like nebulin and nebulin-like proteins (*Neb, Nebl*). Depletion of Tregs during a muscle injury episode delays muscle healing, most probably because of loss of Treg generated amphiregulin. Also, fibro/adipogenic progenitors in skeletal muscles produce high levels of IL33, whose receptor ST2 is highly expressed on muscle Tregs. Thus, muscle Tregs seem to be involved in an alarmin induced repair process (Figure 8A). Interestingly, muscle Treg population declines in old age mice which results in a deterioration of repair and regeneration process (257).

Recently, a very elegant and detailed (258) study in zebrafish has elaborated upon yet unknown and spectacular regenerative capabilities of Tregs (Figure 8B). The authors found that an ortholog of mammalian *Foxp3, Foxp3a*, which was exclusively expressed in a subpopulation of zebrafish T-cells, was upregulated most prominently in distinct regenerating organs. Zebra fish Tregs (zTregs) were predominantly found in kidneys but infiltrated and vigorously proliferated in regenerating tissues. As in the mammalian counterparts, these cells expressed high levels of *Nrp1a* and *Helios* in comparison to kidney zTregs (258). It has been reported that CNS1 region of *Foxp3* locus, responsible for pTreg generation, is not found in zebrafish (259). For identification of zTreg’s role in organ regeneration, punctual and continuous deletion of *Foxp3a* resulted in reduced and delayed regeneration in heart, spinal cord, and retina injury models (258). Deletion of zTregs, in fact, reduced the tissue-specific precursor cells and subdued their proliferation (258). Indeed, zTregs were found near progenitors, sometimes even in close contact (258). However, the most striking finding of this study is that zTregs which presumably came from a common unbiased pool, became plastic in a tissue-specific regenerative context and produced tissue precursor cell specific regeneration factors like Neurotrophin 3 for neural progenitors in regenerating spinal cord, Neuregulin 1 for cardiomyocytes in injured heart and insulin-like growth factor 1 for retinal progenitor Müller glia cells (258) (Figure 8B). That zTregs are the primary source of these growth factors was confirmed by rescue of regeneration in zTreg depleted tissues by recombinant tissue-specific growth factors (258). The regeneration potential of zTregs was independent of their immunosuppressive potential or at least was not dependent on their IL10 production as IL10-deficient cells were fully capable of inducing precursor cell proliferation. However, regeneration potential was *Foxp3a*-dependent as regeneration process was significantly reduced in *Foxp3a*^−^*^/^*^−^ tissues along with growth factor expression levels (258). On the other hand, *Areg* expression was not *Foxp3a* dependent and its role in regeneration was limited. It would be interesting to extrapolate and confirm similar findings in murine and human tissues.

### Feto-Maternal Tolerance

An equally fascinating population of Tregs which accumulates in murine placenta to induce maternal tolerance to fetus has been described (259). To say that the Tregs are extremely important from the outset of pregnancy will not be an overshoot [reviewed in Ref. (260, 261)]. Indeed, mating itself expands uterine Tregs and induces a transient “tolerance” to paternal alloantigens (260). In both humans and mice, seminal plasma contains TGFβ and prostaglandin E, which are potent Treg inducers. In fact, seminal fluid in humans and rodents contains highest measured TGFβ levels among biological fluids (260). Women with recurrent spontaneous abortions have reduced Treg population (262). Female decidual and uterus draining LN Treg generation is CNS1 dependent (259) and increased fetal resorption and placental T-cell infiltration was observed in CNS1-deficient mice. Apart from the their most likely peripheral origin, it is not known whether these Tregs have a distinct phenotypic and functional profile, elucidation of which could come very informative toward amelioration of infertility, pre-eclampsia, and other spontaneous abortive disorders. Very recently, an elegant study on human fetal antigen presenting cells (263) has found that fetal counterparts of DCs are primarily tolerogenic in their response. And, the primary response is generation of Tregs, even more than the adult DCs, in an *in vitro* Treg differentiation assay (263). These DCs were found across several fetal tissues, including spleen, thymus, skin, gut, and lungs (263). Unfortunately, the authors did not describe if Tregs were also present in these tissues. Earlier, it has been shown that human fetal Tregs promote tolerance to non-inherited maternal antigens (264) but only recently, it came to light that Tregs are required for suppression of *in utero* autoimmunity as well. Two children with IPEX syndrome, who died soon after birth, presented histological evidences of tertiary lymphoid structures, chronic inflammatory changes, and targeted exocrine pancreas autoimmunity (265). This signifies that in the perplexing settings of a pregnancy, Tregs are instrumental in establishing tolerance at both ends of maternal–fetal relationship.

## Conclusion and Perspectives

Translational utility of many biological processes is marred by lack of specificity. A similar dilemma exists for Treg biologists as well; however, in case of Tregs selective therapeutic targeting appears to be achievable by virtue of harnessing their gradually established phenotypic and functional diversity. Recent studies have provided evidence that even for the organs like testes and eye, which are conventionally considered immune-privileged; there are populations of Tregs maintaining dominant tolerance and/or tissue homeostasis. While in testis, where otherwise privileged autoantigen escapes from the seminiferous tubules, only to generate systemic tolerance *via* Tregs (266), the retina actively recruits Tregs, which not only attenuate inflammation, but also repair the vasculature, saving blinding neovascular retinopathies (267). Another layer of specificity is added by discovery of tissue-resident Tregs and their unique characteristics. However, most of the information except the recent reports on skin resident Tregs and TI-Tregs are from mouse tissues. There are several differences in structure as well as physiology between mice and humans. For example, mouse skin contains a thin muscle layer *panniculus carnosus*, which is vestigial in humans (268). This helps in contraction, revascularization and healing of wounds without scar formation in mouse. Human skin on the other hand heals by secondary intention leaving scar tissues. Thus, it is important to identify human tissue Tregs for an informed effort toward therapeutic usage.

There is a need to conclusively establish the origin and accumulating factors for tissue Tregs. One of the most pressing questions about almost all the tissue Tregs is identification of their natural ligands or tissue antigens. Although it has been demonstrated that in certain cases Tregs do not need TCR stimulation for some of their functions (150), Tregs with a smaller subset of specific TCR repertoire populate various tissues as well as malignancies. Therefore, cognate ligands that help in survival and proliferation of Tregs in these tissues are likely to have significant contributions in catering tissue-specific modulations. Proof of concept studies provide evidence that Tregs with defined antigen specificity (chimeric antigen receptor Tregs, CAR-Tregs) have potent immunosuppressive functions along with advantage of not inducing generalized immunosuppression (269).

Question remains as to how Tregs communicate with specific tissue cells-like adipocytes to establish a channel of communication with the environment. Beyond adaptation to inflammatory context, there are peculiarities of Treg biology, which modulate their effect temporally in life as well. Such as, Treg accumulation in aging WAT induces insulin resistance (79), whereas its accumulation in young obese WAT ameliorates it (83). The mechanisms that drive such specific outcomes need to be studied in detail. This accentuated capability to adapt sometimes becomes counterproductive too as seen in tumors where the suppressive capacity is enhanced even in comparison to normal tissue-resident Tregs and is, in turn, utilized by tumors for their survival and immune escape. The mechanisms by which Tregs can push the limits of their functional capabilities are yet to be identified.

A major aspect of tissue adaptation is adjusting the cellular metabolism according to the tissue environment. There are huge gaps in our understanding of both lymphoid and tissue Treg metabolism. In *in vitro* differentiated Tregs (iTregs), it was shown that Foxp3 suppresses glycolysis by repression of Myc and helps in developing resistance to l-lactate (270). Similarly, Foxp3 counters PI(3)K-Akt-mTORC1 to diminish glycolysis in iTregs (271). Contrastingly, splenic and TI-Tregs were shown to uptake more 2NBDG, a fluorescent glucose analog, while intratumoral effector T cells showed glucose deprivation leading to reduced production of glycolytic metabolite phosphoenol pyruvate, resulting in compromised effector functions *via* reduced calcium-NFAT signaling (272). More recently, glycolysis was found to be instrumental in Treg trafficking and migration to inflamed tissues. The induction of the glycolytic enzyme glucokinase GCK and cytoskeletal rearrangement upon its association with actin was shown to be critical for the process (273). These findings underscore the need for extensive studies to delineate metabolic reprogramming in not only tissue Tregs but also lymphoid Tregs under steady state and activated conditions.

One can only be amazed by the diversity and functional plasticity of Tregs. A question, therefore, always comes up as to why Tregs are the chosen ones? Whether similar diversities among other immune cell types are still awaiting discoveries, or whether Foxp3 and presumably other unknown factors provide some degree of functional uniqueness to Tregs, remains to be seen. Nevertheless, looking at the diversity of responses ranging from maintaining immune tolerance to tissue repair, to becoming a major stakeholder in maintenance of physiological function of tissues, it would be apt to say that Tregs are the proverbial “Jack of all trades,” and certainly, “master” of some.

## Author Contributions

AS and DR planned and wrote the manuscript.

## Conflict of Interest Statement

The authors declare that the research was conducted in the absence of any commercial or financial relationships that could be construed as a potential conflict of interest.
